# Probing nanomechanical responses of cell membranes

**DOI:** 10.1038/s41598-020-59030-2

**Published:** 2020-02-10

**Authors:** Jichul Kim

**Affiliations:** 1 Institute of Molecular Biology and Genetics, Seoul National University, Seoul, Republic of Korea; 2 Center for Nanomedicine, Institute for Basic Science (IBS) and Yonsei-IBS Institute, Yonsei University, Seoul, Republic of Korea; 3 Research Center for Natural Sciences, Korea Advanced Institute of Science and Technology, Daejeon, Republic of Korea; 4 Department of Mechanical Engineering, Stanford University, Stanford, CA USA

**Keywords:** Membrane biophysics, Nanoscale biophysics

## Abstract

Despite the importance in various cellular processes, the nanomechanical responses of the living cell membrane have been elusive due to complexities in the membrane associated with the hidden architecture of multiple molecular components, including the lipid bilayer. Here, combined experimental and theoretical frameworks that can probe and interpret nanomechanical responses of the cell membrane are demonstrated. A magnetic tweezer assay was introduced to apply pico-Newton scale forces to lipids and E-cadherin molecules at the living cell surface. Two unique classes of force-extension curves were identified: one with a deflection transition (Type I) and another with a discontinuous transition (Type II). The repeated observations of these responses, regardless of cell type and targeted cell surface molecule, suggest the Type I and II curves are the primary nanomechanical responses of cell membranes. To reproduce these responses *in vitro*, a model system using synthetic lipid vesicles was also developed. Together with a finite element model of lipid bilayers, the reproduced responses suggest that the confined fluidity and curvature constraints imposed on the lipid bilayer components of the cell membrane are the main parameters responsible for the generation of these responses. This work provides an insight into how forces on membrane molecules propagate to the lipid bilayer components to generate specific nanomechanical responses. In addition, the consistent results obtained using different methodologies demonstrate that the presented force-probing assays and the theoretical model can serve a combined testbed to investigate nanoscale mechanics of the living cell membrane.

## Introduction

Mechanical forces applied across the cell surface are an important means by which cells communicate with the outside world. Many mechanobiological activities begin with forces applied to membrane-bound receptors responsible for specific cellular tasks. For example, forces applied on adhesion proteins, such as cadherin and integrin, recruit signaling proteins to the cytoplasmic region which influence vital cellular functions, including morphogenesis, migration, and gene expression^1–5^. Those forces on receptor filaments are also known to activate transmembrane channel proteins in order to alter intracellular ionic environments^6,7^.

Less well-appreciated thus far, however, is the fact that forces applied to membrane-bound proteins can also be conveyed to the lipid bilayer membrane itself. Then, the bilayer’s mechanical response can influence the transmission of those mechanical inputs across the cell surface. Membrane tubules are known as micro-mechanical responses of cell membranes observed in numerous past investigations. However, the generation of these tubules can accompany significant modifications of the molecular integrity within the cell surface^8^. Furthermore, their micrometer-scale size and irreversibility hardly support fast and robust propagation of mechanical inputs across the cell surface.

## Results

### Pulling lipids at the living cell surface: observation of Type I and Type II responses

**Figure 1:**
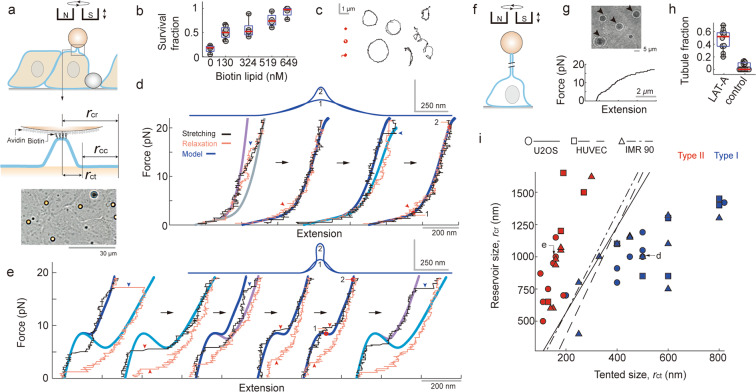
Nanomechanical responses generated by pulling cell surface lipids. (**a**) Schematics for the magnetic tweezer experiments (Top, Middle). Bright-field image for the U2OS sample (Bottom). (**b**) Magnetic bead survival fraction (with ~13 pN in ~15 seconds) with different amounts of biotin-conjugated lipid applied to the cells. About half of the injected beads were still bound to the surface after the force application in the condition of 130 nM biotin lipid. (**c**) Sample traces tracking the center of the magnetic beads as they were rotating with 2–4 pN of pulling force from a magnet. The traces in red show centric rotational motion. (**d**,**e**) Representative force vs. extension curves obtained from successive loading cycles. r_ct_ = 500 nm, r_cr_ = 650 nm (purple); r_ct_ = 500 nm, r_cr_ = 850 nm (gray); r_ct_ = 500 nm, r_cr_ = 1000 nm (dark blue); and r_ct_ = 500 nm, r_cr_ = 1100 nm (blue) were used for calculations in (**d**). r_ct_ = 160 nm, r_cr_ = 1000 nm (dark blue); r_ct_ = 160 nm, r_cr_ = 1500 nm (blue); r_ct_ = 160 nm, r_cr_ = 1170 nm (purple) were used in **e**. The calculated membrane shapes (at red marks) were plotted at the top of (**d**,**e**). (**f**) Schematic for the magnetic tweezer experiments with Latrunculin-A treatments. (**g**) Representative force vs. extension curve with Latrunculin-A. (**h**) The fraction of magnetic beads that generated membrane tubules (see black arrows in **g**) with 20 pN in ~15 seconds. (**i**) Parameterized r_ct_ vs. r_cr_ scatter from multiple bead measurements of three different cell lines (N_U2OS_ = 16, N_HUVEC_ = 10, N_IMR90_ = 19). Measurements with the discontinous transition (Type II) are shown in red and measurements with the kink (Type I) are shown in blue. The decision boundaries (black line) are: −1.0531 + 0.0351r_ct_ − 0.0105r_cr_ = 0; −4.0420 + 0.038r_ct_ − 0.0091r_cr_ = 0; and −1.3143 + 0.0245r_ct_ − 0.0063r_cr_ = 0 for U2OS, HUVEC, and IMR90, respectively.

To this end, nanomechanical responses of cell membranes were investigated using magnetic tweezers^9,10^. Human bone osteosarcoma epithelial cells (U2OS)^5,11,12^ were seeded and cultured until they formed an epithelial monolayer on a substrate. Then, biotin-conjugated lipids were treated and washed to attach magnetic beads to the apical surface of the cultured cells via biotin-avidin binding (Fig. 1a,b, see Supplementary Fig. S1 for the cellular viability test). Here, beads with greater thermal fluctuations were more frequently observed by reducing the treatment of the biotin-conjugated lipids, which may suggest the reduced bead-surface interaction with the treatment of smaller amounts of the biotin lipids (Supplementary Fig. S2a).

Before making force response measurements for cells incubated with ~65–260 nM of the biotinylated lipids, both the natural fluctuation and induced rotation of the magnetic beads were checked. Beads with minimal fluctuation and wider or non-circular rotations with severe wobbling, which may indicate poor surface targeting, were not sampled during magnetic tweezer experiments (Fig. 1c). Note that sliding of beads on the cell surface was occasionally observed during rotation. Finally, the force-extension curves were recorded by pulling the biotin-conjugated lipids with a constant loading rate and tracking the vertical position of the magnetic beads.

A lot of complexity were observed in the measured responses, including both vertical motion and lateral shifting of the beads even without significant force application. In repeated experiments, however, two types of nanoscale responses with unique mechanistic features were identified. These responses were distinct from the classic tubular structures formed at the cell membrane. Figure 1d shows one representative force-extension curve (referred to as Type I) in the second and fourth loading cycles. This type of force-extension curve starts with a compliant regime and exhibits a biphasic character organized around a deflection or kink in the curve. Figure 1e shows another type of force-extension curve (hereafter referred to as Type II). Without the noticeable deflection, the second, fourth, and fifth cycles of the responses start with an initial stiff barrier and feature a discontinuous transition (i.e. unstable stretching of the membrane) to another stiff regime. In many cases, a small sign for the discontinuous re-transition was also observed during the relaxation. Finally, the microscopic movement of the magnetic beads was observed when the cells were treated with Latrunculin-A. The force-extension response demonstrated the micro-scale extraction of the cell membrane (Fig. 1f–h). Since Latrunculin-A is known to inhibit actin polymerization^13^, the result suggests an intact cytoskeletal network is important for the generation of Type I and II nanomechanical responses.

### Mathematical model of nanomechanical lipid bilayers: comparison between measurements and calculations

Next, whether theories of lipid bilayer mechanics can explain the Type I and II measurements was investigated. To this end, a simple mathematical model of the nanomechanical lipid bilayer that combines a well-known curvature elasticity theory with an expression for membrane surface tension was introduced^14–17^. The total energy functional in domain Ω Ψ_*nano membrane*_ was formulated as in Eq. (1).

1 $${\Psi }_{nanomembrane}=\mathop{\int }\limits_{\Omega }\,(2{k}_{m}{H}^{2}+{k}_{g}K)dA+{\int }_{{\alpha }_{0}}^{{\alpha }_{c}}\,\sigma d\alpha \cdot \mathop{\int }\limits_{\Omega }dA$$

The first term integrates the energy densities due to the mean (*H*) and Gaussian curvatures (*K*) over the membrane area (*A*). Here *k*_m_ and *k*_g_ denote the bending and Gaussian curvature moduli of the membrane, respectively. The second term incorporates the free energy change due to area strain of the membrane. The surface tension (σ) is the function of the area strain of the membrane $\alpha =\frac{({{A}}^{{res}}-{A}_{0}^{{res}})}{{A}_{0}^{{res}}}=\frac{({{\rm{\phi }}}_{0}^{{res}}-{{\rm{\phi }}}^{{res}})}{{{\rm{\phi }}}^{{res}}}$ where *A*^*res*^ is the area of the lipid membrane reservoir determined by r_cr_ - r_ct_, Ω, and r_cb_ (see the next paragraph and Fig. S3 in Supplementary Information online); ${A}_{0}^{{res}}$ is the area of the lipid reservoir at the resting reference configuration; ${{\rm{\phi }}}^{{res}}$ is the uniform lipid number density; and ${{\rm{\phi }}}_{0}^{{res}}$ is ${{\rm{\phi }}}^{{res}}$ at the resting reference configuration. The function integration of σ with respect to α gives the surface strain energy density. Finite element modeling for this theory was performed, whose full description is shown in the Methods (also see an approach in ref. ^18^). The estimates of shape and applied force resulting from the application of a specific displacement of the lipid bilayer can be calculated using this model.

Note the presence of geometric parameters in this model (Fig. 1a): the radius of the lipid reservoir that defines the limit of lateral membrane stretching (r_cr_) and the radius of the area where the membrane is tightly associated with the rigid cytoskeleton (r_cc_). From r_cr_ and r_cc_, the radius of the membrane tented (r_ct_ = r_cr_ − r_cc_) can be defined. In the rotational axisymmetric configuration, these simple parameters provide a conceptual idea on the physical boundaries responsible for the generation of the nanomechanical responses. Remarkably, with an assumption of point-like pulling, these model-based membrane predictions showed a reasonable fit to the Type I and II observational data. For the Type I data presented in the second and fourth cycles of Fig. 1d, r_ct_ = 500 nm and r_cr_ = 1000 nm were used to fit the calculation to the measurements. Without introducing additional parameter values, a reasonable fit for the Type II data was also achieved by modulating the r_ct_ and generating a sigmoidal-type curve. With a fixed r_cr_, reducing r_ct_ resulted in the generation of the initial barrier for the force vs. extension calculation. For the fourth and fifth cycle of Fig. 1e, r_ct_ = 160 nm and r_cr_ = 1000 nm were used.

In many cases, using more than one r_cr_ values provided a better interpretation for the single force vs. extension measurement. An additional r_cr_ value (i.e. another limit of the lipid reservoir) was introduced to explain a secondary transition (see blue arrows) followed by the deflection in the first and third cycles of Fig. 1d, and the discontinuous transition in the first, third, and sixth cycles of Fig. 1e (also see other examples in supplementary Fig. S5e). Four r_cr_ values with a shared r_ct_ of 500 nm were used for all four successive cycles of measurements in Fig. 1d. Similarly, three r_cr_ values with a single r_ct_ of 160 nm were used for all six successive cycles of measurements in Fig. 1e.

While measurements were sorted into Type I or Type II responses, a more detailed examination of their force-extension shapes did show some variation. By using the lipid bilayer model, a diagram of Type I and II measurements was generated for Fig. 1i. The data in this diagram were smoothly separated in the r_ct_-r_cr_ plane, depending on the type of measured force-extension responses. These results suggest that the simple model reasonably explains not only the individual force vs. extension trace but also the whole data set on a higher level. There are also limitations in comparison between the model and the observation. According to the model, for example, beads attached with non-negligible membrane area can also generate Type I and II nanomechanical responses, providing a possibility of an underestimation for the r_ct_ and r_cr_ values (see Supplementary Fig. S3 for the discussion). In addition, same material parameters for the membrane were used for all calculations while they may vary for different region of the real cell surface (see Supplementary Table S1). Nevertheless, predictions made by using theories of lipid bilayers seem to indicate that the bilayer’s elastic properties determined by r_ct_ and r_cr_ are important factors for the generation of Type I and II responses. Of note, r_ct_ values in the diagram were similar to the size of membrane compartments determined from imaging molecular hop diffusion^19,20^. Studying whether these share a common biophysical underpinning might be informative.

### Pulling lipids at the surface of different cell types

Lipid bilayers are a common factor of the membrane, regardless of the type of the cell. To test observed Type I and II responses in other cell types beyond U2OS cells, the same pulling protocol was applied to living human umbilical vein endothelial cells (HUVEC) and human fetal lung fibroblasts (IMR-90). Because these cell types are distinct from U2OS cells, they may have different underlying cellular substructures. Nevertheless, both Type I and II responses were also observed when pulling the lipids of these cell types. In addition, the model similarly explained these measurements (Supplementary Figs. S4 and S5). While the Type I and II data fell in slightly different regions of the r_ct_-r_cr_ diagram, the decision boundaries determined for each respective cell line were largely overlapping (Fig. 1i). The result suggests a similar principle of membrane nanomechanics for different cell types, even if their predominant targeting response might be different.

### *In vitro* model of synthetic nanovesicles: repnroduction of Type I and Type II responses

**Figure 2:**
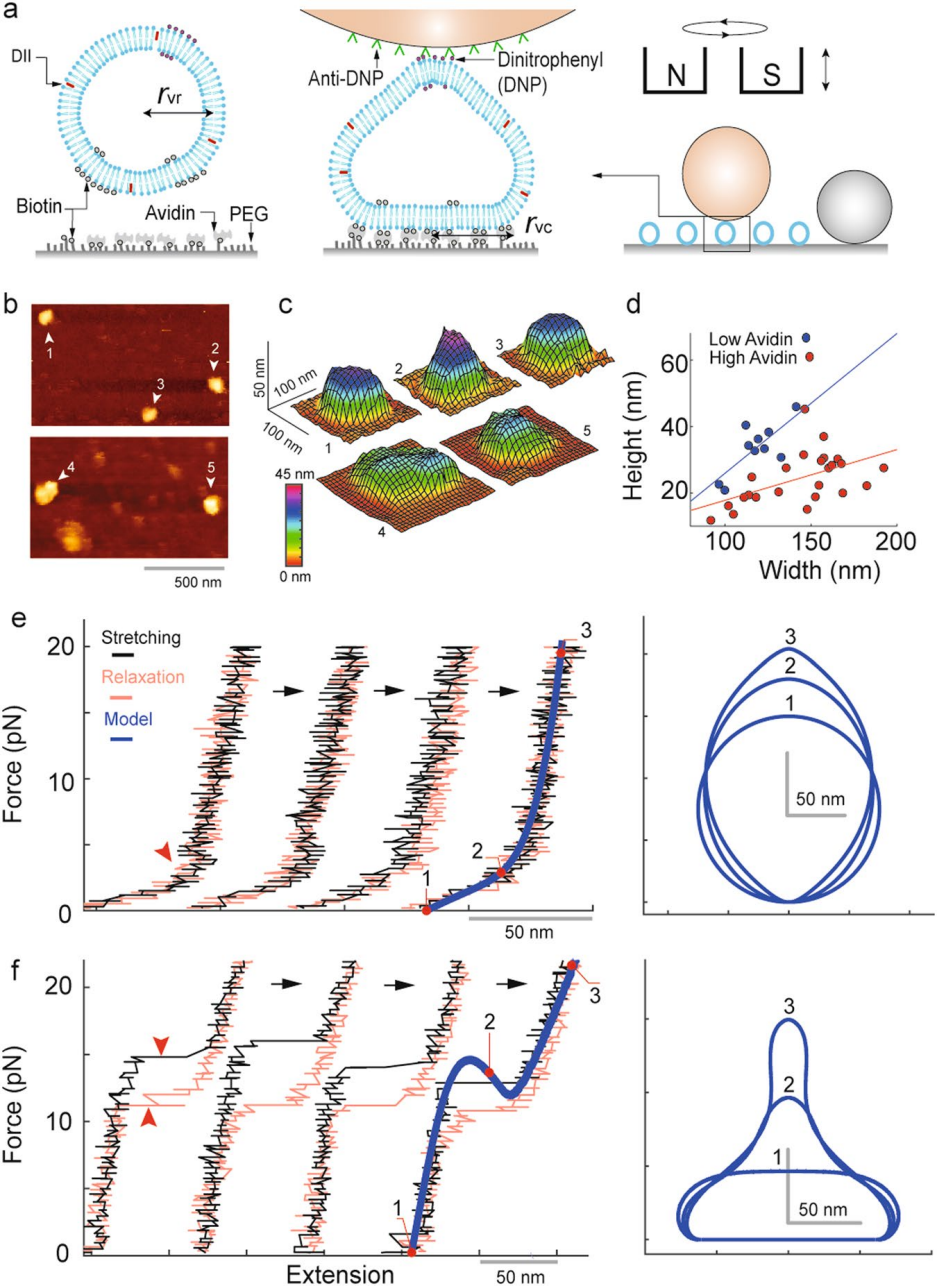
Reproduced nanomechanical responses from synthetic lipid vesicles. (**a**) Schematics for the magnetic tweezer experiments with synthetic lipid vesicles. (**b**) Atomic force microscopy (AFM) Quantitative Imaging (QI) of vesicles with different levels of avidin density (top: lower avidin density, bottom: higher avidin density) on a glass substrate. (**c**) The reconstructed shape of the vesicles in (**b**). (**d**) Vesicle height vs. width scatter obtained from the AFM images with the two different surface conditions. (**e**) Representative force vs. extension measurements obtained from a single vesicle in successive loading cycles in the lower avidin condition. The calculation was fitted for the measurements, and the corresponding vesicle shapes at three extension points were plotted. r_vr_ = 75 nm and r_vc_ = 1 nm were used. (**f**) Successive force vs. extension measurements from a vesicle in the higher avidin condition. r_vr_ = 62 nm and r_vc_ = 62 nm were used for the calculation.

The observed Type I and II nanomechanical responses, likely generated from the lipid bilayer components of living cell membranes, were reproduced *in vitro*. To this end, synthetic lipid vesicles with a mean diameter of 118 ± 14 (S.D.) nm were made via an extrusion method (Fig. 2d, see Supplementary Information online). These nanoscale vesicles have a limited size of lipid reservoir. The vesicles were immobilized on a glass substrate using biotin-avidin bonding. Magnetic beads were then attached to single vesicles via minimally treated dinitrophenyl-conjugated lipids as demonstrated in Fig. 2a. Before beginning the pulling protocol, rotatory motion of the beads was checked as similarly done for the cell membrane. With these nanovesicles, remarkably, the Type I force-extension response was reproduced as shown in Fig. 2e (also see Supplementary Fig. S6a–c).

**Figure 3:**
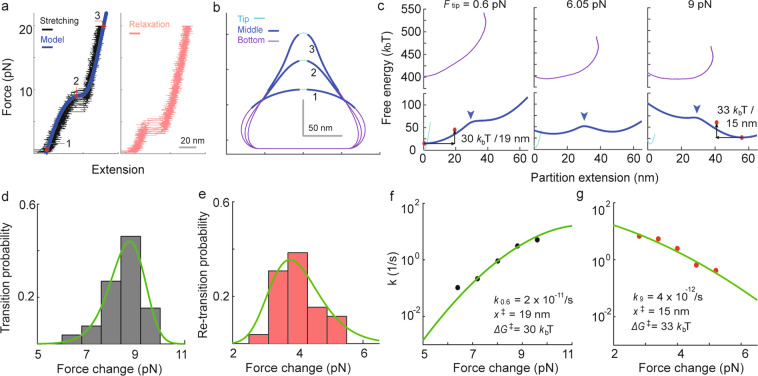
Energy analyses for the bi-stable response of lipid vesicles. (**a**) Force vs. extension cycles repeated multiple times (N_repeat_ = 26) for a single vesicle. The calculation used r_vr_ = 70 nm and r_vc_ = 58 nm. (**b**) The calculated vesicle shapes (corresponding to the red marks in **a**) were divided into three partitions. (**c**) Energy vs. extension calculations for each of the three partitions in (**b**) with different levels of tip force (colors are matched). Energy barriers are indicated by blue arrows. See Supplementary Fig. S8 for the detailed calculations of the free energy of each partition. (**d**,**e**) Transition and re-transition force histograms obtained from data in (**a**). (**f**,**g**) The kinetic rate plots correspond to (**d**,**e**), respectively. Fitting the data with a transition kinetic theory (green curves in **d**–**g**) provides information about the free energy barrier (Δx^ǂ^, ΔG^ǂ^, k _force change_) of the transition and retransition. See Supplementary Information online for the details of the analysis.

Analysis performed for the cell membrane measurements suggested increased curvature constraints (i.e., increased r_cc_ or decreased r_ct_) can generate the Type II response. To validate this idea, greater levels of rigid body interaction was imposed on the immobilized vesicles by simply increasing the biotin-avidin interactions between the vesicles and the underlying surface. A higher concentration of avidins increased the area of attachment, which led to a flattening of the immobilized vesicles (Fig. 2b–d, see Supplementary Information online). Finally, when these vesicles of higher avidin condition were pulled, the Type II response was more frequently observed than the Type I (Figs. 2f and 3a, also see Supplementary Fig. S6d,e).

The reproduced vesicle responses were also interpreted with the finite element model (see Methods). The energy functional is expressed in Eq. (2).

2 $${\varPsi }_{nanovesicle}=\mathop{\int }\limits_{\varOmega }\,(2{k}_{m}{H}^{2}+{k}_{g}K)dA+{\int }_{{\alpha }_{0}}^{{\alpha }_{c}}\,\sigma d\alpha \cdot \mathop{\int }\limits_{\varOmega }\,dA+\lambda (\mathop{\int }\limits_{\varOmega }\,dV-{V}_{0})$$

The last term with the Lagrange multiplier (λ) reflects the condition of the fixed volume (*V*_0_) spanned by the vesicle. Here, the two geometric parameters introduced for the cell membrane, r_cr_ and r_cc_, were replaced with r_vr_ (the vesicle radius in a deformation-free configuration) and r_vc_ (the radius of the area of contact with the underlying surface). Figure 2e,f show that calculations with reasonably defined r_vr_ and r_vc_ values explain both Type I and II responses of vesicles (also see Supplementary Fig. S6). The force decomposition analysis shown in Supplementary Fig. S7 provides an interpretation of these biphasic responses^21^. According to the analysis, the initial force-extension curve is dominated by the force due to the membrane curvature development. The deflection (Fig. S7a) and the re-rise of the curve after the turning point (Fig. S7b) coincide with the point where the force due to membrane stretching becomes significant. The analysis suggests that additional membrane curvature development, to prevent the membrane strain energy from becoming excessive, is responsible for the responses in the larger extension regime.

With a particular interest in the bi-stable system, how the energy landscape of Type II responses interprets the observed discontinuous transitions was investigated in Fig. 3. For a vesicle where Type II force-extension curves were measured from sufficiently repeated loading cycles, the shape was calculated and divided into three partitions (Fig. 3a,b, see Supplementary Information online). Then, the energy of these three partitions (i.e., tip, middle, and bottom partitions) was calculated by taking the vesicle height as a generalized coordinate (Fig. 3c, see Supplementary Fig. S8 and Supplementary Information online). Even with nearly zero tension, the energy landscape of the middle partition showed two minima separated by an energy barrier (Fig. 3c blue arrows). As the tension was increased, the local minimum with a larger extension was shifted downward, becoming a global minimum at around 9 pN (Fig. 3c). Calculations suggest that the transition between these two energy minima appears as the step extension in the Type II force vs. extension curves.

The energy landscape was also evaluated by using a theory widely used in single-molecule force spectroscopy^22^. The membrane calculation suggests that the change of the generalized force to the middle partition is nearly the same with the tip force within the force range examined. Accordingly, transition and re-transition force histograms for the middle partition were generated from magnetic force measurements (Fig. 3a,d,e), where the energy barrier information was estimated (Fig. 3d–g, see Supplementary Information online). The results of this analysis were consistent with the estimates of the membrane model (Fig. 3c red marks). Together, the highly controlled measurements and subsequent multidisciplinary analyses performed with synthetic lipid vesicles reveal the biophysical principles that produce the novel mechanical responses of the cell membrane, which include bistability.

### Pulling E-cadherins at the living cell surface

**Figure 4:**
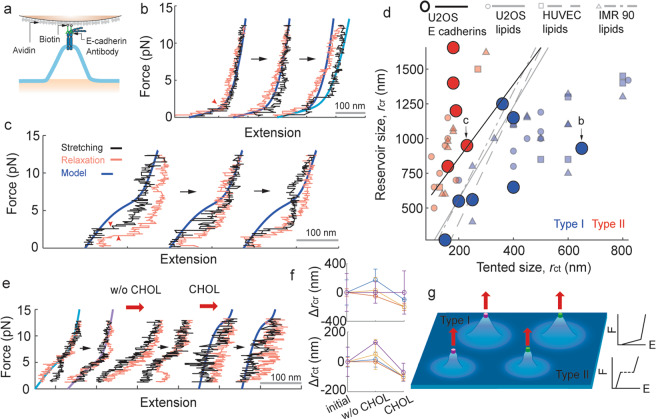
Nanomechanical responses generated by pulling cell surface E-cadherins. (**a**) Schematic for magnetic tweezer experiments pulling E-cadherins. (**b**,**c**) Representative force vs. extension curves obtained from successive loading cycles. r_ct_ = 650 nm, r_cr_ = 930 nm (dark blue) and r_ct_ = 650 nm, r_cr_ = 1200 nm (blue) in (**b**); r_ct_ = 230 nm, r_cr_ = 950 nm in (**c)** were used. (**d**) r_ct_ vs. r_cr_ scatter (N_bead_ = 12). The decision boundary is 2.1785 + 0.0176r_ct_ − 0.0066r_cr_ = 0. Data points from Fig. 1i are also shown for comparison. (**e**) Representative force vs. extension curves in successive cycles of force with treatment of a buffer of cholesterol saturated with 20 mM methyl-β-cyclodextrin. Magnetic force was held at 15 pN during buffer flow. Minimum holding force during the cycles is 1.3 pN. r_ct_ = 200 nm, r_cr_ = 600 nm for blue; r_ct_ = 220 nm, r_cr_ = 600 nm for purple; and r_ct_ = 110 nm, r_cr_ = 500 nm for dark blue were used. (**f**) Median values for change of r_ct_ and r_cr_ after buffer exchange (N_bead_ = 4). (**g**) A mechanism of force transmission in the cell membrane. Force (red arrows) applied on different membrane molecules (magenta and green) is conveyed to generate Type I or Type II nanomechanical force (*F*) vs. extension (*E*) responses of the lipid bilayer (blue).

Force is transmitted to the cell surface, in many cases, through membrane receptor proteins. To gain collective insight on how these receptor forces are conveyed to the lipid bilayer, the experimental and theoretical methodologies were also applied to pulling E-cadherin proteins expressed endogenously on the surface of living U2OS cells. Here, the membrane proximal ectodomains of the cadherins were targeted with biotinylated E-cadherin antibodies (Fig. 4a, Supplementary Figs. S2b and S9)^23,24^, so any possible contributions from the extracellular cadherin repeats would be minimized^25^. Notably, force-extension curves with the deflection and the discontinuous transition were also observed in repeated experiments pulling E-cadherins (Fig. 4b,c). Furthermore, when these responses were examined by using the lipid bilayer model, the individual force-extension curves could be fitted with similar r_ct_ and r_cr_ values as for the lipid pulling experiments. Finally, the scatter of these values in the r_ct_-r_cr_ diagram largely overlaps with the data obtained from the lipid pulling experiments (Fig. 4d). These results suggest the remarkable possibility that lipid bilayer responses can be triggered by the pulling of the E-cadherin. Although it is unclear whether and how the cadherin and any associated molecules contribute to the observed responses, the results demonstrate that mechanical pulling on a membrane-bound protein could produce direct displacements of the lipid bilayer.

To further support the idea of bilayer responses generated via force on a membrane-bound protein, a buffer of cholesterol saturated in 20 mM methyl-β-cyclodextrin (MβCD) was treated while pulling E-cadherin (Fig. 4e). First, the magnetic beads that showed the nanomechanical responses were selected and treated with pure buffer injections. Next, the beads were asked whether similar force-extension responses can be produced even after the buffer flow. In many cases, the force-extension shapes were altered after the flow, likely due to structural changes of the perturbed cell membrane. There were cases, however, without critical modifications in the force vs. extension responses. When these beads were further treated with the cholesterol-MβCD buffer, changes in curves were observed within several cycles of pulling and relaxation of the cadherin. These changes were explained by a decrease of r_ct_ or r_cr_ within the presented model framework (Fig. 4e,f). Cholesterol-MβCD complexes are known to enrich the membrane cholesterol level^26,27^, suggesting that cholesterol is inducing this reduction. Cholesterols are known to modulate rigidity and fluidity of the lipid bilayer^28^. The idea that the changes in the nanomechanical responses are due to changes in the lipid bilayer itself is well-supported by the functional and biochemical properties of membrane cholesterols.

## Discussion

Numerous cellular events begin with force applied across the cell membrane. In addition to asking for how applied forces are transduced into a certain cellular phenotype^2,5,29^, an important subject in recent studies of mechanobiological processes is to investigate how the mechanical responses propagate within the force-bearing elements^30^. Detailed mechanical responses had been identified for various systems at the single-molecular level *in vitro*^25,31–35^. However, the nanomechanical responses of cell membranes that can serve an important role in the propagation of mechanical energy across the cell surface had been largely elusive.

Here, by targeting cell surface molecule lipids and E-cadherins, two primary nanomechanical responses of living cell membranes were identified, one with a deflection (Type I) and the other with a discontinuous transition (Type II) of the force-extension curve. These responses were universal regardless of tested cell type and targeted molecule in the cell membrane. *In vitro* vesicle and theoretical analyses performed for the observed responses suggested that, while force receptors can vary on the cell surface, the shape of the mechanical responses can be determined by the lipid bilayer components in which the molecule is inserted (Fig. 4g). The modulation of the fluidity and flexibility of the lipid bilayer are important determinants of the properties of these responses. Questions remain to be answered for how cells take advantage or disadvantage of these unique mechanical characteristics of the lipid bilayer for specific physiological functions. Overall, the work provides standardized living, synthetic, and theoretical frameworks to study nanoscale mechanics of the cell membrane. Furthermore, the work demonstrates how mechanical cues can propagate from the receptor proteins to the lipid bilayer components to contribute to nanomechanical responses of the cell membrane, and thus provides a coupled force transmission paradigm for various processes at the cell surface.

## Methods

### Finite element modeling for lipid membranes

#### Weak form of the problem

Finite element methods for the membrane theory are provided here in detail. Since Eqs. (1) and (2) share the identical formalisms without the third term of Eq. (2), descriptions are adapted for Eq. (2). As shown in the results section, the total energy functional in (2) is expressed with three terms: (1) the Canham-Helfrich curvature energy term, (2) a simple area strain energy term, and (3) a term for the fixed volume V_0_ with a Lagrange multiplier λ. Note that λ = 0 can be defined for Eq. (1).

2 $${\varPsi }_{nanovesicle}=\mathop{\int }\limits_{\varOmega }\,(2{k}_{m}{H}^{2}+{k}_{g}K)dA+{\int }_{{\alpha }_{0}}^{{\alpha }_{c}}\,\sigma d\alpha \cdot \mathop{\int }\limits_{\varOmega }\,dA+\lambda (\mathop{\int }\limits_{\varOmega }\,dV-{V}_{0})$$

The differential forms of the surface are described using the Monge gauge with respect to the parametric coordinate of the model. The mean curvature H and Gaussian curvature K for the rotational axisymmetric membrane geometry can be expressed as follows^36,37^

3 $${\rm{H}}=0.5(\frac{{{\rm{h}}}_{{\rm{rr}}}}{{\sqrt{1+{{\rm{h}}}_{{\rm{r}}}^{2}}}^{3}}+\frac{{{\rm{h}}}_{{\rm{r}}}}{{\rm{r}}\sqrt{1+{{\rm{h}}}_{{\rm{r}}}^{2}}})\,{\rm{and}}\,{\rm{K}}=\frac{{{\rm{h}}}_{{\rm{rr}}}{{\rm{h}}}_{{\rm{r}}}}{{\rm{r}}(1+{{\rm{h}}}_{{\rm{r}}}^{2}{)}^{2}}$$

here, the function h measures the height of the lipid membrane with respect to the radial function r. Since the model describes vesicle shape in a parametric domain s[0,1], the first and second derivatives of the membrane height h with respect to r, which are indicated by h_r_ and h_rr_, can be expressed through parametric derivatives by h_r_ = h_s_/r_s_ and h_rr_ = h_ss_/r_s_^2^ − h_s_r_ss_/r_s_^3^, respectively. The expressions ${\rm{dA}}=2\pi r\sqrt{{h}_{s}^{2}+{r}_{s}^{2}}ds$ and ${\rm{dV}}=\pi {r}^{2}$(−*h*_*s*_)*ds* are the vesicle axisymmetric area and volumetric elements, respectively. The constants k_m_ and k_g_ in Eq. (2) are the bending and Gaussian curvature modulus of the membrane, respectively. Here k_g_ = 0.5 k_m_ is used. An expression for the membrane surface tension σ was determined from Eq. (4)^16,17^

4 $$\begin{array}{c}{\rm{\sigma }}={{\rm{\sigma }}}_{0}\cdot \exp (\frac{8{{\rm{\pi }}{\rm{k}}}_{{\rm{m}}}}{{{\rm{k}}}_{{\rm{b}}}{\rm{T}}}{\rm{\alpha }})\,{\rm{for}}\,{\rm{\alpha }}\le {{\rm{\alpha }}}_{{\rm{cross}}}\\ {\rm{\sigma }}={{\rm{K}}}_{{\rm{app}}}({\rm{\alpha }}-{{\rm{\alpha }}}_{{\rm{cut}}})\,{\rm{for}}\,{\rm{\alpha }} > {{\rm{\alpha }}}_{{\rm{cross}}}\end{array}$$

here, the cut-off strain α_cut_ and cross-over strain α_cross_ are defined to have smooth continuity for the two surface tension expressions in Eq. (4) at α_cross_. The constant σ_0_ is surface tension with zero strain, and K_app_ is the apparent area stretching modulus. The vesicle area strain is $\alpha =\frac{({{A}}^{{res}}-{A}_{0}^{{res}})}{{A}_{0}^{{res}}}=\frac{({{\rm{\phi }}}_{0}^{{res}}-{{\rm{\phi }}}^{{res}})}{{{\rm{\phi }}}^{{res}}}$ where ${{A}}^{{res}}$ is the area of the vesicle membrane determined by r_vc_, Ω, and r_cb_ (see Fig. S3 in Supplementary Information online); ${A}_{0}^{{res}}$ is the resting reference area of the vesicle in the spherical configuration; ${{\rm{\phi }}}^{{res}}$ is the uniform lipid number density; and ${{\rm{\phi }}}_{0}^{{res}}$ is ${{\rm{\phi }}}^{{res}}$ at the resting reference configuration. Therefore, the function integration of surface tension with respect to the area strain α from initial zero strain α_0_ to the strain under consideration α_c_ estimates surface strain energy density as denoted in the second term of Eq. (2). See Supplementary Table S1 for the membrane parameters used in this study.

In the variational context, finite element solutions can be found by taking the first variation of Eq. (2) equal to zero–a condition necessary to minimize free energy. To this end, the variational equation is defined in (5) as follows:

5 $${\rm{\delta }}{\varPsi }_{nanovesicle}=\mathop{\int }\limits_{\Omega }\,({{\rm{A}}{\rm{\delta }}{\rm{h}}}_{{\rm{ss}}}+{{\rm{B}}{\rm{\delta }}{\rm{h}}}_{{\rm{s}}}+{{\rm{C}}{\rm{\delta }}{\rm{r}}}_{{\rm{ss}}}+{{\rm{D}}{\rm{\delta }}{\rm{r}}}_{{\rm{s}}}+{\rm{E}}{\rm{\delta }}{\rm{r}}+{\rm{F}}{\rm{\delta }}{\rm{\lambda }}){\rm{ds}}=0$$

where ${\rm{A}}=\frac{\partial {{\rm{\psi }}}_{H}}{\partial {{\rm{h}}}_{{\rm{ss}}}}$, ${\rm{B}}=\frac{\partial ({{\rm{\psi }}}_{H}+{{\rm{\psi }}}_{\lambda })}{\partial {{\rm{h}}}_{{\rm{s}}}}+\frac{2{T}_{{\rm{\alpha }}}\pi r{h}_{s}}{\sqrt{{h}_{s}^{2}+{r}_{s}^{2}}}$, ${\rm{C}}=\frac{\partial {{\rm{\psi }}}_{H}}{\partial {{\rm{r}}}_{{\rm{ss}}}}$, ${\rm{D}}=\frac{\partial {{\rm{\psi }}}_{H}}{\partial {{\rm{r}}}_{{\rm{s}}}}+\frac{2{T}_{{\rm{\alpha }}}\pi r{r}_{s}}{\sqrt{{h}_{s}^{2}+{r}_{s}^{2}}}$, ${\rm{E}}=\frac{\partial ({{\rm{\psi }}}_{H}+{{\rm{\psi }}}_{\lambda })}{\partial {\rm{r}}}+2{T}_{{\rm{\alpha }}}\pi \sqrt{{h}_{s}^{2}+{r}_{s}^{2}}$, and ${\rm{F}}=\frac{\partial {{\rm{\psi }}}_{\lambda }}{\partial {\rm{\lambda }}}$. Here ${{\rm{\psi }}}_{H}$ is integrand associated with the curvature energy and ${{\rm{\psi }}}_{\lambda }$ is integrand for the term of volumetric constraints when Eq. (2) is expanded with respect to parametric domain s. The expression T_α_ is defined as derived in the Appendix A in Supplementary Information online. Here, the Gaussian curvature energy contribution can be omitted because surface integration of the Gaussian curvature is invariant for the axisymmetric deformation. Therefore, their variations are identically zero, according to the Gauss-Bonnet theorem^38^. The functions δh and δs denote variations of the membrane shape functions h and r, respectively.

Since Eq. (5) is expressed by the maximum second order of the membrane shape functions, the collection of trial solutions S and admissible variations V with boundary conditions up to its first derivative are defined as follows:

6 $$\begin{array}{c}{\rm{S}}=\{({\rm{h}},{\rm{r}})\,{\rm{smooth}}|{\rm{h}}(0)={{\rm{h}}}_{0},{{\rm{h}}}_{{\rm{s}}}(0)={{\rm{h}}}_{{\rm{s}},0},{\rm{h}}(1)={{\rm{h}}}_{1},{{\rm{h}}}_{{\rm{s}}}(1)={{\rm{h}}}_{{\rm{s}},1},{\rm{r}}(0)\\ \,={{\rm{r}}}_{0},{{\rm{r}}}_{{\rm{s}}}(0)={{\rm{r}}}_{{\rm{s}},0},{\rm{r}}(1)={{\rm{r}}}_{1},{{\rm{r}}}_{{\rm{s}}}(1)={{\rm{r}}}_{{\rm{s}},1}\}\end{array}$$

and

7 $${\rm{V}}=\{({\rm{\delta }}{\rm{h}},{\rm{\delta }}{\rm{r}})\,{\rm{smooth}}\,|{\rm{\delta }}{\rm{h}}(0)={\rm{\delta }}{\rm{h}}(1)={{\rm{\delta }}{\rm{h}}}_{{\rm{s}}}(0)={{\rm{\delta }}{\rm{h}}}_{{\rm{s}}}(1)={\rm{\delta }}{\rm{r}}(0)={\rm{\delta }}{\rm{r}}(1)={{\rm{\delta }}{\rm{r}}}_{{\rm{s}}}(0)={{\rm{\delta }}{\rm{r}}}_{{\rm{s}}}(1)=0\}$$

here, h_0_ and r_0_ are the prescribed displacements at s = 0, as well as h_s,0_ and r_s,0_ are the prescribed slope at s = 0. Similarly, h_1_, r_1_, and h_s,1_, r_s,1_ are the prescribed displacements and slopes at s = 1. Finally, the weak form of the presented boundary value problem can be stated, “Find (h, r) ∈ S and λ ∈ ${\mathbb{R}}$ satisfying Eq. (5) for ∀(δh, δr) ∈ V and ∀δλ ∈ ${\mathbb{R}}$”.

#### Galerkin form

The Galerkin form of the problem can be stated by approximating function spaces S (6) and V (7) in the weak statement to the finite dimensional space S^h^ and V^h^. Here, the parameterized functions for membrane shape (h^h^, r^h^) and their variation (δh^h^, δr^h^) belong to S^h^ and V^h^, respectively, i.e., (h^h^, r^h^) ∈ S^h^ ⊂ S and (δh^h^, δr^h^) ∈ V^h^ ⊂ V. Therefore, the Galerkin approximated solution of Eq. (5) can be found by solving (8) as follows:

8 $${\rm{\delta }}{\varPsi }^{{\rm{h}}}=\mathop{\int }\limits_{\Omega }\,{{\rm{\delta }}{\rm{\psi }}}^{{\rm{h}}}{\rm{ds}}={\int }_{\Omega }({{\rm{A}}}^{{\rm{h}}}{{\rm{\delta }}{\rm{h}}}_{{\rm{ss}}}^{{\rm{h}}}+{{\rm{B}}}^{{\rm{h}}}{{\rm{\delta }}{\rm{h}}}_{{\rm{s}}}^{{\rm{h}}}+{{\rm{C}}}^{{\rm{h}}}{{\rm{\delta }}{\rm{r}}}_{{\rm{ss}}}^{{\rm{h}}}+{{\rm{D}}}^{{\rm{h}}}{{\rm{\delta }}{\rm{r}}}_{{\rm{s}}}^{{\rm{h}}}+{{\rm{E}}}^{{\rm{h}}}{{\rm{\delta }}{\rm{r}}}^{{\rm{h}}}+{{\rm{F}}}^{{\rm{h}}}{\rm{\delta }}{\rm{\lambda }}){\rm{ds}}=0$$

where ${{\rm{\delta }}{\rm{\psi }}}^{{\rm{h}}}={{\rm{\delta }}{\rm{\psi }}({\rm{h}}}_{{\rm{ss}}}^{{\rm{h}}},{{\rm{h}}}_{{\rm{s}}}^{{\rm{h}}},{{\rm{r}}}_{{\rm{ss}}}^{{\rm{h}}},{{\rm{r}}}_{{\rm{s}}}^{{\rm{h}}},{{\rm{r}}}^{{\rm{h}}},{\rm{\lambda }})$. With Eq. (8), it is required to assume a function u^h^ that belongs to the space V^h^ (i.e., u^h^ ∈ V^h^ ⊂ V), and then define the function h^h^ with respect to u^h^ by introducing a function w^h^ that satisfies the boundary conditions in S, i.e., w^h^(0) = h_0_, w^h^_s_(0) = h_s,0_, w^h^(1) = h_1_ and w^h^_s_(1) = h_s,1_. Here, the functions h^h^, u^h^, and w^h^ are related as follows: ${{\rm{h}}}^{{\rm{h}}}={{\rm{u}}}^{{\rm{h}}}+{{\rm{w}}}^{{\rm{h}}}$. By similarly introducing a function v^h^ and x^h^ for r^h^ where ${{\rm{r}}}^{{\rm{h}}}={{\rm{v}}}^{{\rm{h}}}+{{\rm{x}}}^{{\rm{h}}}$, the Galerkin approximation for the weak form can be stated: “Find (u^h^, v^h^) ∈ V^h^ and λ ∈ ${\mathbb{R}}$ satisfying Eq. (8) for ∀(δh^h^, δr^h^) ∈ V^h^ and ∀δλ ∈ ${\mathbb{R}}$.”

#### B-spline-based approximation

With the given Galerkin form of the problem, the structure of the functions δh^h^, δr^h^, u^h^, and v^h^ in space V^h^, as well as the structure of the given functions w^h^ and x^h^ that satisfy the boundary conditions need to be defined. Since δh^h^, δr^h^, u^h^, and v^h^ belong to H^2^ functions, C^1^ conforming elements are required. The model parameterizes axisymmetric membrane shape with parametric B-spline functions. Although quadratic functions are used here, the numerical framework introduced in this work can be extended for any type of spline function family.

Instead of parameterizing δh^h^ and δr^h^ (and u^h^ and v^h^) independently to provide full two-dimensional degrees of freedom (DOFs) for the finite element nodes, the motion of each node was constrained into the one-dimensional normal direction of a given reference curve. As previously discussed, such treatment was effective for avoiding the so-called zero-energy mode in numerical methods for Helfrich-type membrane models^18^. Since the model solves the shape of the membrane by applying infinitesimal displacement steps during nonlinear calculations, here, the reference curve was simply defined from the membrane shape calculated in the previous displacement step.

Based on the approach summarized above, the parameterized variation of the shape δh^h^ and δr^h^ can be expressed as follows by setting that a spline basis function N(s)_i_ is associated with the i^th^ node in the discretized parametric coordinate s.

9 $${{\rm{\delta }}{\rm{h}}}^{{\rm{h}}}=\mathop{\sum }\limits_{{\rm{i}}=1}^{{\rm{n}}}\,{{\rm{N}}}_{{\rm{i}}}{{\rm{c}}}_{{\rm{i}}}\,\sin \,{{\rm{\theta }}}_{{\rm{i}}}$$

and

10 $${{\rm{\delta }}{\rm{r}}}^{{\rm{h}}}=\mathop{\sum }\limits_{{\rm{i}}=1}^{{\rm{n}}}\,{{\rm{N}}}_{{\rm{i}}}{{\rm{c}}}_{{\rm{i}}}\,\cos \,{{\rm{\theta }}}_{{\rm{i}}}$$

here, c_1_ to c_n_ are unknown values, and θ_1_ to θ_n_ define the normal direction for the DOFs with respect to the given reference curve.

To model the parameterized shape function h^h^, w^h^ is defined as in Eq. (11) using the same spline basis function N.

11 $${{\rm{w}}}^{{\rm{h}}}={{\rm{N}}}_{-1}{{\rm{dh}}}_{-1}+{{\rm{N}}}_{0}{{\rm{dh}}}_{0}+{{\rm{N}}}_{{\rm{n}}+1}\,{{\rm{dh}}}_{{\rm{n}}+1}+{{\rm{N}}}_{{\rm{n}}+2}\,{{\rm{dh}}}_{{\rm{n}}+2}$$

Since δh^h^ and u^h^ belong to the same function space V^h^, h^h^ is defined as follows (see Supplementary Fig. S10 for the visualization of the parameterization):

12 $${{\rm{h}}}^{{\rm{h}}}={{\rm{u}}}^{{\rm{h}}}+{{\rm{w}}}^{{\rm{h}}}={{\rm{N}}}_{-1}\,{{\rm{dh}}}_{-1}+{{\rm{N}}}_{0}{{\rm{dh}}}_{0}+\mathop{\sum }\limits_{{\rm{i}}=1}^{{\rm{n}}}\,{{\rm{N}}}_{{\rm{i}}}({{\rm{d}}}_{{\rm{i}}}\,\sin \,{{\rm{\theta }}}_{{\rm{i}}}+{{\rm{dh}}}_{{\rm{ref}}.,{\rm{i}}})+{{\rm{N}}}_{{\rm{n}}+1}\,{{\rm{dh}}}_{{\rm{n}}+1}+{{\rm{N}}}_{{\rm{n}}+2}\,{{\rm{dh}}}_{{\rm{n}}+2}$$

where

13 $${{\rm{u}}}^{{\rm{h}}}=\mathop{\sum }\limits_{{\rm{i}}=1}^{{\rm{n}}}\,{{\rm{N}}}_{{\rm{i}}}({{\rm{d}}}_{{\rm{i}}}\,\sin \,{{\rm{\theta }}}_{{\rm{i}}}+{{\rm{dh}}}_{{\rm{ref}}.,{\rm{i}}})$$

In Eqs. (11) and (12), N_−1_, N_0_, N_n+1_, and N_n+2_ are B-spline basis functions for the boundary region, and dh_−1_, dh_0_, dh_n+1_, and dh_n+2_ are the given fixed values that define the boundary conditions. d_1_ to d_n_ are unknown values. The constant values dh_ref.,1_ to dh_ref.,n_ are obtained from the reference configuration. Similarly, r^h^ can be defined as follows with dr_−1_, dr_0_, dr_n+1_, dr_n+2_, and dr_ref.,1_ to dr_ref.,n_.

14 $${{\rm{r}}}^{{\rm{h}}}={{\rm{N}}}_{-1}\,{{\rm{dr}}}_{-1}+{{\rm{N}}}_{0}{{\rm{dr}}}_{0}+\mathop{\sum }\limits_{{\rm{i}}=1}^{{\rm{n}}}\,{{\rm{N}}}_{{\rm{i}}}({{\rm{d}}}_{{\rm{i}}}\,\cos \,{{\rm{\theta }}}_{{\rm{i}}}+{{\rm{dr}}}_{{\rm{ref}}.,{\rm{i}}})+{{\rm{N}}}_{{\rm{n}}+1}\,{{\rm{dr}}}_{{\rm{n}}+1}+{{\rm{N}}}_{{\rm{n}}+2}\,{{\rm{dr}}}_{{\rm{n}}+2}$$

The first and second derivatives with respect to s can be derived from the given structure of δh^h^, h^h^, δr^h^, and r^h^. Substituting those into Eq. (8) as well as the arbitrariness of c_i_ in Eqs. (9) and (10) and δλ results in coupled n + 1 nonlinear simultaneous equations, as denoted by the vector notation in (15).

15 $$\bar{{\rm{G}}}=[\begin{array}{c}[{{\rm{G}}}_{{\rm{a}}}]\\ {{\rm{G}}}_{{\rm{n}}+1}\end{array}]$$

Here, the residual vector $\bar{{\rm{G}}}$ has a total of n + 1 rows with its a^th^ row component G_a_ (for 1 ≤ a ≤ n) being defined in Eq. (16). There, each equation contains five nodal unknowns in using the quadratic B-spline basis function.

16 $${{\rm{G}}}_{{\rm{a}}}={\int }_{{\Omega }_{{\rm{a}}}}{[\begin{array}{l}\mathop{{\rm{A}}}\limits^{\frown {}}\\ \mathop{{\rm{B}}}\limits^{\frown {}}\\ \mathop{{\rm{C}}}\limits^{\frown {}}\\ \mathop{{\rm{D}}}\limits^{\frown {}}\\ \mathop{{\rm{E}}}\limits^{\frown {}}\end{array}]}^{{\rm{T}}}[\begin{array}{l}{{\rm{N}}}_{{\rm{ss}}}{({\rm{s}})}_{{\rm{a}}}\,\sin \,{{\rm{\theta }}}_{{\rm{a}}}\\ {{\rm{N}}}_{{\rm{s}}}{({\rm{s}})}_{{\rm{a}}}\,\sin \,{{\rm{\theta }}}_{{\rm{a}}}\\ {{\rm{N}}}_{{\rm{ss}}}{({\rm{s}})}_{{\rm{a}}}\,\cos \,{{\rm{\theta }}}_{{\rm{a}}}\\ {{\rm{N}}}_{{\rm{s}}}{({\rm{s}})}_{{\rm{a}}}\,\cos \,{{\rm{\theta }}}_{{\rm{a}}}\\ {\rm{N}}{({\rm{s}})}_{{\rm{a}}}\,\cos \,{{\rm{\theta }}}_{{\rm{a}}}\end{array}]{\rm{ds}}=0$$

Here, $\mathop{{\rm{A}}}\limits^{\frown {}}$, $\mathop{{\rm{B}}}\limits^{\frown {}}$, $\mathop{{\rm{C}}}\limits^{\frown {}}$, $\mathop{{\rm{D}}}\limits^{\frown {}}$ and $\mathop{{\rm{E}}}\limits^{\frown {}}$ represent parameterized A^h^, B^h^, C^h^, D^h^, and E^h^ in Eq. (8), respectively. The n + 1^th^ element of the residual vector that is associated with a Lagrange multiplier can be defined as follows:

17 $${{\rm{G}}}_{{\rm{n}}+1}=\mathop{\int }\limits_{\Omega }\,[\mathop{{\rm{F}}}\limits^{\frown {}}]{\rm{ds}}=0$$

Here, similarly, $\mathop{{\rm{F}}}\limits^{\frown {}}$ represents the parameterized F^h^ in Eq. (8).

#### Linearization: jacobian matrix for the newton–raphson method

Given n + 1 nonlinear equations in (15), a tangential operator (i.e., n + 1 by n + 1 Jacobian matrix) can be derived to use Newton’s method to iteratively obtain solutions of the nonlinear equation system (i.e., d_1_, d_2_, …, d_n_ and λ). For this purpose, the Jacobian matrix $\bar{{\rm{J}}}$ of the residual vector $\bar{{\rm{G}}}$ is defined as follows:

18 $$\overline{{\rm{J}}}=[\begin{array}{ll}[{{\rm{j}}}_{{\rm{a}},{\rm{b}}}] & [{{\rm{j}}}_{{\rm{a}},{\rm{n}}+1}]\\ {{\rm{j}}}_{{\rm{n}}+1,{\rm{b}}} & 0\end{array}]$$

Here, the elements in the a^th^-row and the b^th^-column of the matrix (for 1 ≦ a ≦ n, 1 ≦ b ≦ n) can be found from Eq. (19) (see Appendix B in Supplementary Information online).

19 $$\begin{array}{rcl}{{\rm{j}}}_{{\rm{a}},{\rm{b}}} & = & \frac{\partial {{\rm{G}}}_{{\rm{a}}}}{\partial {{\rm{d}}}_{{\rm{b}}}}\,{\rm{for}}\,-2\le {\rm{b}}-{\rm{a}}\le 2\\  & = & 0\,{\rm{otherwise}}\end{array}$$

By assuming the infinitesimal lateral oscillation of the membrane area (also see next paragraph), T_α_ (see Appendix A in Supplementary Information online) is assumed to be constant in expanding Eq. (19), which provides great simplicity. The elements of n-by-1 $[{{\rm{j}}}_{{\rm{a}},{\rm{n}}+1}]$ and 1-by-n $[{{\rm{j}}}_{{\rm{n}}+1,{\rm{b}}}]$ matrices can be obtained as ${{\rm{j}}}_{{\rm{a}},{\rm{n}}+1}=\frac{\partial {{\rm{G}}}_{{\rm{a}}}}{\partial {\rm{\lambda }}}$ and ${{\rm{j}}}_{{\rm{n}}+1,{\rm{b}}}=\frac{\partial {{\rm{G}}}_{{\rm{n}}+1}}{\partial {{\rm{d}}}_{{\rm{b}}}}$, respectively.

Finally, by substituting an initial guess for the solutions ${\bar{{\rm{d}}}}_{0}$ (i.e. d_1_, d_2_, …, d_n_ and λ) to the Jacobian matrix $\bar{{\rm{J}}}$ and the residual vector $\bar{{\rm{G}}}$, the solution vector $\bar{{\rm{d}}}$ for j + 1^th^ Newton’s iteration can be calculated from ${\bar{{\rm{d}}}}_{{\rm{j}}+1}={\bar{{\rm{d}}}}_{{\rm{j}}}-\bar{{\rm{J}}}{({\bar{{\rm{d}}}}_{{\rm{j}}})}^{-1}\bar{{\rm{G}}}({\bar{{\rm{d}}}}_{{\rm{j}}})$. Here fixed values for the $\bar{{\rm{d}}}$ were substituted into the terms associated with the area strain T_α_ to avoid the potent numerical oscillation and thus the divergence of the iterative process. The strain value for k^th^ displacement step was predicted from k − 1^th^ step and corrected for k + 1^th^ step. Such treatments might be supported by assuming the infinitesimal lateral oscillation of the membrane area from the outside of the parametric domain for different displacement steps. To account some large deformation of the cell membrane, the infinitesimal shifting of the r_cr_ was also allowed to satisfy a condition that the tented membrane area of the k^th^ stretching step is greater than or equal with that of the k − 1^th^ step and by assuming the curvature deformation limit. Gaussian quadrature and standard mapping techniques were used to compute element arrays for the Jacobian matrix $\bar{{\rm{J}}}$ and the residual vector $\bar{{\rm{G}}}$. With given boundary values, this iterative process is continued until the Euclidean norm of the difference of two subsequent solution vectors converges to a certain tolerance. See Supplementary Information and Algorithm S1 online for simulation details.

## Experimental Methods

The mechanical pulling experiments were carried out using a previously developed magnetic tweezer apparatus^9,10^. After introducing polystyrene reference beads to the bottom surface of the channel slides purchased from ibidi, cells were seeded and cultured on the slides until they formed an epithelial monolayer. Then, streptavidin-coated magnetic beads (Dynabeads® M-280 streptavidin) were introduced to the upper surface of the cellular layer after treatment and washing of ~65–260 nM biotinylated lipids (Fig. 1), and ~0.5 μg/ml of biotinylated E-Cadherin monoclonal antibody (Fig. 4). The force vs. extension responses were measured with a constant loading rate in 2.1–2.6 pN/s. For the cholesterol flow experiments, a sufficient amount of cholesterol powders was mixed with 20 mM methyl-β-cyclodextrin. After overnight incubation at 37 °C with vortexing, undissolved cholesterol remains were filtered from the buffer. This cholesterol solution was applied with a flow velocity of 3.3 ul/sec during the buffer exchange process.

For the vesicle experiments, a channel of ~20 μl volume where the bottom coverslip was coated with polyethylene glycol (PEG) polymer chains was constructed. Two types of PEG-coated coverslips were prepared with two different ratios used in mixing the PEG polymer chains and the biotin-conjugated PEG polymer chains. Vesicles were made by following a typical extrusion protocol^39,40^ using polycarbonate membrane filters with 100 nm pores. The anti-dinitrophenyl antibody was conjugated to the magnetic beads (Dynabeads® M-270 Carboxylic Acid) to target dinitrophenyl-conjugated lipids in the vesicle. Atomic force microscopic images for the vesicle were acquired with NanoWizard Ultra Speed (JPK Instruments) in Quantitative Imaging (QI) mode. Triangular Si_3_N_4_ cantilevers with the spring constant of 0.02 N/m were used. Both vesicle and live-cell experiments were performed at room temperature (22–25 °C). See Supplementary Information online for experimental method details.

## Supplementary information

Supplementary Information.
