# Author Correction: Probing nanomechanical responses of cell membranes

**DOI:** 10.1038/s41598-020-80518-4

**Published:** 2021-01-14

**Authors:** Jichul Kim

**Affiliations:** 1 Institute of Molecular Biology and Genetics, Seoul National University, Seoul, Republic of Korea; 2 Center for Nanomedicine, Institute for Basic Science (IBS) and Yonsei-IBS Institute, Yonsei University, Seoul, Republic of Korea; 3 Research Center for Natural Sciences, Korea Advanced Institute of Science and Technology, Daejeon, Republic of Korea; 4 Department of Mechanical Engineering, Stanford University, Stanford, CA USA

Correction to: *Scientific Reports* 10.1038/s41598-020-59030-2, published online 10 February 2020

The original version of this Article contained errors.

In the Results section under subheading ‘Mathematical model of nanomechanical lipid bilayers: comparison between measurements and calculations’,

“The surface tension ($\upsigma$) is a function of the area strain (${\upalpha }$) of the membrane (${\upalpha }=\left(\mathrm{A}-{\mathrm{A}}_{0}\right)/{\mathrm{A}}_{0}$) with a resting area of ${\mathrm{A}}_{0}$, and the function integration of $\upsigma$ with respect to ${\upalpha }$ gives the surface strain energy density.”

now reads:

“The surface tension ($\upsigma$) is the function of the area strain of the membrane ${\upalpha }=\frac{\left({A}^{res}-{A}_{0}^{res}\right)}{{A}_{0}^{res}}=\frac{\left({\upphi }_{0}^{res}-{\upphi }^{res}\right)}{{\upphi }^{res}}$ where ${A}^{res}$ is the area of the lipid membrane reservoir determined by ${\mathrm{r}}_{\mathrm{cr}}$-${\mathrm{r}}_{\mathrm{ct}}$, $\Omega$, and ${\mathrm{r}}_{\mathrm{cb}}$ (see the next paragraph and Fig. S3 in Supplementary Information online); ${A}_{0}^{res}$ is the area of the lipid reservoir at the resting reference configuration; ${\upphi }^{res}$ is the uniform lipid number density; and ${\upphi }_{0}^{res}$ is ${\upphi }^{res}$ at the resting reference configuration. The function integration of $\upsigma$ with respect to ${\upalpha }$ gives the surface strain energy density.”

In the Methods section under subheading ‘Finite element modeling for lipid membranes’,

“The vesicle area strain is ${\upalpha }=\left(\mathrm{A}-{\mathrm{A}}_{0}\right)/{\mathrm{A}}_{0}= \left({\upphi }_{0}-\upphi \right)/\upphi$ with resting (i.e., initial) area ${\mathrm{A}}_{0}$ or initial lipid density ${\upphi }_{0}$.”

now reads:

“The vesicle area strain is ${\upalpha }=\frac{\left({A}^{res}-{A}_{0}^{res}\right)}{{A}_{0}^{res}}=\frac{\left({\upphi }_{0}^{res}-{\upphi }^{res}\right)}{{\upphi }^{res}}$ where ${A}^{res}$ is the area of the vesicle membrane determined by ${\mathrm{r}}_{\mathrm{vc}}$, $\Omega$, and ${\mathrm{r}}_{\mathrm{cb}}$ (see Fig. S3 in Supplementary Information online); ${A}_{0}^{res}$ is the resting reference area of the vesicle in the spherical configuration; ${\upphi }^{res}$ is the uniform lipid number density; and ${\upphi }_{0}^{res}$ is ${\upphi }^{res}$ at the resting reference configuration.”

16 $${\mathrm{G}}_{\mathrm{a}}={\int }_{{\Omega }_{\mathrm{a}}}\left[\begin{array}{c}{\overset{\lower0.5em\hbox{$\smash{\scriptscriptstyle\frown}$}}{\text{A}}}\\ {\overset{\lower0.5em\hbox{$\smash{\scriptscriptstyle\frown}$}}{\text{B}}}\\ {\overset{\lower0.5em\hbox{$\smash{\scriptscriptstyle\frown}$}}{\text{C}}}\\ {\overset{\lower0.5em\hbox{$\smash{\scriptscriptstyle\frown}$}}{\text{D}}}\\ {\overset{\lower0.5em\hbox{$\smash{\scriptscriptstyle\frown}$}}{\text{E}}}\end{array}\right]\left[\begin{array}{c}{\mathrm{N}}_{\mathrm{ss}}{\left(\mathrm{s}\right)}_{\mathrm{a}}\mathrm{sin}{\uptheta }_{\mathrm{a}}\\ {\mathrm{N}}_{\mathrm{s}}{\left(\mathrm{s}\right)}_{\mathrm{a}}\mathrm{sin}{\uptheta }_{\mathrm{a}}\\ {\mathrm{N}}_{\mathrm{ss}}{\left(\mathrm{s}\right)}_{\mathrm{a}}\mathrm{cos}{\uptheta }_{\mathrm{a}}\\ {\mathrm{N}}_{\mathrm{s}}{\left(\mathrm{s}\right)}_{\mathrm{a}}\mathrm{cos}{\uptheta }_{\mathrm{a}}\\ \mathrm{N}{\left(\mathrm{s}\right)}_{\mathrm{a}}\mathrm{cos}{\uptheta }_{\mathrm{a}}\end{array}\right]\mathrm{ds}=0$$

now reads:

16 $${\mathrm{G}}_{\mathrm{a}}={\int }_{{\Omega }_{\mathrm{a}}}{\left[\begin{array}{c}{\overset{\lower0.5em\hbox{$\smash{\scriptscriptstyle\frown}$}}{\text{A}}}\\ {\overset{\lower0.5em\hbox{$\smash{\scriptscriptstyle\frown}$}}{\text{B}}}\\ {\overset{\lower0.5em\hbox{$\smash{\scriptscriptstyle\frown}$}}{\text{C}}}\\ {\overset{\lower0.5em\hbox{$\smash{\scriptscriptstyle\frown}$}}{\text{D}}}\\ {\overset{\lower0.5em\hbox{$\smash{\scriptscriptstyle\frown}$}}{\text{E}}}\end{array}\right]}^{\mathrm{T}}\left[\begin{array}{c}{\mathrm{N}}_{\mathrm{ss}}{\left(\mathrm{s}\right)}_{\mathrm{a}}\mathrm{sin}{\uptheta }_{\mathrm{a}}\\ {\mathrm{N}}_{\mathrm{s}}{\left(\mathrm{s}\right)}_{\mathrm{a}}\mathrm{sin}{\uptheta }_{\mathrm{a}}\\ {\mathrm{N}}_{\mathrm{ss}}{\left(\mathrm{s}\right)}_{\mathrm{a}}\mathrm{cos}{\uptheta }_{\mathrm{a}}\\ {\mathrm{N}}_{\mathrm{s}}{\left(\mathrm{s}\right)}_{\mathrm{a}}\mathrm{cos}{\uptheta }_{\mathrm{a}}\\ \mathrm{N}{\left(\mathrm{s}\right)}_{\mathrm{a}}\mathrm{cos}{\uptheta }_{\mathrm{a}}\end{array}\right]\mathrm{ds}=0$$

“For this purpose, a symmetric and positive-definite Jacobian matrix $\overline{\text J}$ of the residual vector $\overline{G}$ is defined as follows:”

now reads:

“For this purpose, the Jacobian matrix $\overline{\text J}$ of the residual vector $\overline{G}$ is defined as follows:”

“Based on a general pursuit of finer elements in the methods, terms of ${\text{G}}_{\text{a}}$ associated with the membrane area strain ${\upalpha }$ i.e., ${\text{T}}_{\upalpha }$ (see Appendix A in Supplementary Information online) are assumed to be constant values, which provides great simplicity for expanding Eq. (19).”

now reads:

“By assuming the infinitesimal lateral oscillation of the membrane area (also see next paragraph), ${\text{T}}_{\upalpha }$ (see Appendix A in Supplementary Information online) is assumed to be constant in expanding Eq. (19), which provides great simplicity.”

“Finally, by substituting an initial guess for the solutions $\overline{\text{d}}_{0}$ (i.e. d_1_, d_2_, …, d_n_ and λ) to the Jacobian matrix $\overline{\text{J}}$ and the residual vector $\overline{\text{G}}$, the solution vector $\overline{\text{d}}$ for j + 1^th^ Newton’s iteration can be calculated from $\overline{\text d}_{{\text j} + 1} = \overline{\text d}_{\text j} + \cdot \overline{\text J}\left( {\overline{\text d}_{\text j} } \right)^{ - 1} \cdot \overline{\text G}\left( {\overline{\text d}_{\text j} } \right).$

now reads:

“Finally, by substituting an initial guess for the solutions $\overline{\text{d}}_{0}$ (i.e. d_1_, d_2_, …, d_n_ and λ) to the Jacobian matrix $\overline{\text{J}}$ and the residual vector $\overline{\text{G}}$, the solution vector $\overline{\text{d}}$ for j + 1^th^ Newton’s iteration can be calculated from $\overline{\text d}_{{\text j} + 1} = \overline{\text d}_{\text j} - \overline{\text J}\left( {\overline{\text d}_{\text j} } \right)^{ - 1} \overline{\text G}\left( {\overline{\text d}_{\text j} } \right)$”

“With given boundary values, this iterative process is continued until the difference of two subsequent solution vectors converges to a certain tolerance.”

now reads:

“With given boundary values, this iterative process is continued until the Euclidean norm of the difference of two subsequent solution vectors converges to a certain tolerance.”

These errors have now been corrected in the PDF and HTML versions of the Article.

