# Supplementary material for: Probing nanomechanical responses of cell membranes
Source: Sci Rep. 2020 Feb 10;10:2301. doi: 10.1038/s41598-020-59030-2 (PMC7010710; doi:10.1038/s41598-020-59030-2)
Supplement: Supplementary file 1 — Supplementary Information. [file 41598_2020_59030_MOESM1_ESM.docx]

Supplementary Information for

**Probing nanomechanical responses of cell membranes**

Jichul Kim^1,2,3,4^

^1^Institute of Molecular Biology and Genetics, Seoul National University, Seoul, Republic of Korea

^2^Center for Nanomedicine, Institute for Basic Science (IBS) and Yonsei-IBS Institute, Yonsei University, Seoul, Republic of Korea

^3^Research Center for Natural Sciences, Korea Advanced Institute of Science and Technology, Daejeon, Republic of Korea

^4^Department of Mechanical Engineering, Stanford University, Stanford, CA, USA

1. **Finite element simulation**

**Point force**

An explicit formula for the point force can be defined in the finite element framework. The applied point force at s = 0 to the direction of h can be derived from following equation (20).

$f_{0}=\frac{\partial G_{F.E.}}{\partial h_{0}}=\int_{\Omega} \left[ A\frac{\partial h_{\mathrm{ss}}}{\partial h_{0}}+B\frac{\partial h_{s}}{\partial h_{0}}+C\frac{\partial r_{\mathrm{ss}}}{\partial h_{0}}+D\frac{\partial r_{s}}{\partial h_{0}}+E\frac{\partial r}{\partial h_{0}} \right]\mathrm{ds}$ (20)

G_F.E._ represents a stationary of the$\Psi$ in equation (2). Note that the originally given boundary values dh_-1_ and dh_0_ should be treated as unknown variables in the derivation of equation (20). The given boundary values and the solution for the nodal values (i.e. d_1_, ···, d_n_) can then be substituted into (20) to obtain the point force.

**Simulation for substrate immobilization and point pulling of the nanovesicle**

There are two main parts in the single vesicle finite element simulation. The first part mimics substrate immobilization of the vesicle. For this purpose, the simulation initially starts with a vesicle with a perfect spherical shape and then prescribes the location of the boundary at s = 1 with infinitesimal increments to make the vesicle an oblate spheroid contacting the glass bottom. Here, the contact area and initial radius of the vesicle are indicated with r_vc_ and r_vr_, respectively (see diagram in the main text). Membrane surface tension for each displacement step was calculated and the desired volume of the vesicle, i.e., V_0_ in equation (2) was adjusted when the calculated tension was greater than a pre-defined value. This process represents a possible leakage of intra-vesicular solutions due to area stretching during long-range incubation in real experiments. Here, the prescribed boundary values at s = 1 were adjusted (with trial and error) to have f_0_≈0 pN and σ≈σ_0_ at the end of the immobilization simulation. When simulating pulling a spherical vesicle (i.e., a point-like immobilization), these processes can be omitted.

The second part of the simulation was performed by prescribing boundary values at s = 0 to modulate tip displacement of the vesicle. Although the desired vesicle volume was altered in the immobilization simulation, it was assigned a fixed value during the pulling process.

**Vesicle partitioning**

An algorithm is introduced to define membrane partitions where the number of lipids is preserved in pulling the vesicle. The algorithm defines the sectioned membrane partitions by satisfying the following relation for each displacement step k and for each i^th^ partition element:

$\frac{A_{i,k}}{A_{tot,k}}=Const_{i}$ for ∀i and ∀k (21)

A_i,k_ and A_tot,k_ are the area of rotational axisymmetric i^th^ partition and the total area of the vesicle, respectively, for the k^th^ displacement step. Here, the constant values Const_i_ were defined to have an arc-length of reasonably uniform size for the sectioned partitions. By using this partitioning algorithm, it was possible to calculate the energy of the sectioned membrane with a fixed lipid number during mechanical deformation of the vesicle.

**Energy vs. extension of the vesicle partition with forces**

Post-process analyses were performed to visualize the energy vs. extension curves for each vesicle partition. To plot the energy landscape of the membrane partitions in the presence of a specific tip force, the generalized force of the partitions corresponding to a specific tip displacement value was calculated. The derivative of the partition energy (G_F.E.P._) with respect to partition extension (x) by varying vesicle-tip displacement was taken to calculate the generalized force acting on each vesicle partition. To provide the smooth continuity for the discrete numerical data, the G_F.E.P_ and x were fitted to Fourier series by using a built-in function ‘fourier8’ in Matlab (Mathworks®). This generalized force value f was then used to plot the partition energy with f (G_f_) vs. partition extension (x) relationship through

$G_{f}=G_{F.E.P.}-f\left( x-x_{f} \right)$. (22)

Here, G_F.E.P,_, which is a function of x, represents stationary partition energy directly calculated from the finite element model. The value x_f_ provides a reference extension with f, shown with black dots in Fig. S8.

**Appendix A: variational equations for the membrane strain energy**

From equation (4), the strain energy density shown in equation (1) and (2) can be written as shown in equation (23)

$$\int_{\alpha_{0}}^{\alpha} \sigma d\alpha=\frac{\sigma_{0}}{c_{1}}exp\left( c_{1}\alpha\right)-\frac{\sigma_{0}}{c_{1}} for \alpha\leq\alpha_{\mathrm{cross}}$$

$\int_{\alpha_{0}}^{\alpha} \sigma d\alpha=\frac{K_{app}}{2}\alpha^{2}-K_{app}\alpha_{cut}\alpha+c_{2} for \alpha>\alpha_{\mathrm{cross}}$ (23)

where

$$\alpha=\frac{A}{A_{0}}-1=\frac{\phi_{0}}{\phi}-1$$

$$c_{1}=\frac{8\pi k_{m}}{k_{b}T}$$

and

$c_{2}=\frac{\sigma_{0}}{c_{1}}exp\left( c_{1}\alpha_{cross} \right)-\frac{\sigma_{0}}{c_{1}}-\left( \frac{K_{app}}{2}\alpha_{cross}^{2}-K_{app}\alpha_{cut}\alpha_{cross} \right)$

Therefore, the variational form of the strain energy term ${\delta\Psi}_{\alpha} \mathrm{for}\alpha\leq\alpha_{\mathrm{cross}}$can be derived as follow.

$${\delta\Psi}_{\alpha}=\delta\left[ \left( \frac{\sigma_{0}}{c_{1}}exp\left( c_{1}\left( \frac{A}{A_{0}}-1 \right) \right)-\frac{\sigma_{0}}{c_{1}} \right)A \right]$$

$$=\left[ \frac{\sigma_{0}A}{A_{0}}exp\left( c_{1}\left( \frac{A}{A_{0}}-1 \right) \right)+\frac{\sigma_{0}}{c_{1}}exp\left( c_{1}\left( \frac{A}{A_{0}}-1 \right) \right)-\frac{\sigma_{0}}{c_{1}} \right]\delta A$$

$=T_{\alpha}\delta\left( \int2\pi r\sqrt{h_{s}^{2}+r_{s}^{2}}ds \right)$

where

$T_{\alpha}=\left[ \frac{\sigma_{0}\phi_{0}}{\phi}exp\left( c_{1}\alpha\right)+\frac{\sigma_{0}}{c_{1}}exp\left( c_{1}\alpha\right)-\frac{\sigma_{0}}{c_{1}} \right]$ (24)

Similarly, the variational equation of the strain energy for $\alpha>\alpha_{\mathrm{cross}}$ is

$${\delta\Psi}_{\alpha}=\delta\left[ \left( \frac{K_{app}}{2}\left( \frac{A}{A_{0}}-1 \right)^{2}-K_{app}\alpha_{cut}\left( \frac{A}{A_{0}}-1 \right)+c_{2} \right)A \right]$$

$$=\left[ \frac{K_{app}A}{A_{0}}\left( \frac{A}{A_{0}}-1 \right)-\frac{K_{app}\alpha_{cut}A}{A_{0}}+0.5K_{app}\left( \frac{A}{A_{0}}-1 \right)^{2}-K_{app}\alpha_{cut}\left( \frac{A}{A_{0}}-1 \right)+c_{2} \right]\delta A$$

$=T_{\alpha}\delta\left( \int2\pi r\sqrt{h_{s}^{2}+r_{s}^{2}}ds \right)$

where

$T_{\alpha}=\left[ \frac{K_{app}\phi_{0}}{\phi}\alpha-\frac{K_{app}\alpha_{cut}\phi_{0}}{\phi}+0.5K_{app}\alpha^{2}-K_{app}\alpha_{cut}\alpha+c_{2} \right]$ (25)

**Appendix B: further expansion of equation (19)**

$$j_{a,b}=\frac{\partial G_{a}}{\partial d_{b}}=\int_{\Omega_{a}} \left( \left[ \begin{matrix} \frac{\partial A}{\partial h_{ss}^{h}} & \frac{\partial A}{\partial h_{s}^{h}} & \frac{\partial A}{\partial r_{ss}^{h}} & \frac{\partial A}{\partial r_{s}^{h}} & \frac{\partial A}{\partial r^{h}} \\ \frac{\partial B}{\partial h_{ss}^{h}} & \frac{\partial B}{\partial h_{s}^{h}} & \frac{\partial B}{\partial r_{ss}^{h}} & \frac{\partial B}{\partial r_{s}^{h}} & \frac{\partial B}{\partial r^{h}} \\ \frac{\partial C}{\partial h_{ss}^{h}} & \frac{\partial C}{\partial h_{s}^{h}} & \frac{\partial C}{\partial r_{ss}^{h}} & \frac{\partial C}{\partial r_{s}^{h}} & \frac{\partial C}{\partial r^{h}} \\ \frac{\partial D}{\partial h_{ss}^{h}} & \frac{\partial D}{\partial h_{s}^{h}} & \frac{\partial D}{\partial r_{ss}^{h}} & \frac{\partial D}{\partial r_{s}^{h}} & \frac{\partial D}{\partial r^{h}} \\ \frac{\partial E}{\partial h_{ss}^{h}} & \frac{\partial E}{\partial h_{s}^{h}} & \frac{\partial E}{\partial r_{ss}^{h}} & \frac{\partial E}{\partial r_{s}^{h}} & \frac{\partial E}{\partial r^{h}} \end{matrix} \right]\left[ \begin{matrix} \frac{\partial h_{ss}^{h}}{\partial{dh}_{b}}\sin\theta_{b} \\ \frac{\partial h_{s}^{h}}{\partial{dh}_{b}}\sin\theta_{b} \\ \frac{\partial r_{ss}^{h}}{\partial{dr}_{b}}\cos\theta_{b} \\ \frac{\partial r_{s}^{h}}{\partial{dr}_{b}}\cos\theta_{b} \\ \frac{\partial r^{h}}{\partial{dr}_{b}}\cos\theta_{b} \end{matrix} \right] \right)^{T}\left[ \begin{matrix} N_{\mathrm{ss}}\left( s \right)_{a}\sin\theta_{a} \\ N_{s}\left( s \right)_{a}\sin\theta_{a} \\ N_{\mathrm{ss}}\left( s \right)_{a}\cos\theta_{a} \\ N_{s}\left( s \right)_{a}\cos\theta_{a} \\ N\left( s \right)_{a}\cos\theta_{a} \end{matrix} \right]ds$$

$$for -2\leq b-a\leq2$$

$= 0 otherwise$ (19)

where ${{dh}_{b}=d}_{b}\sin\theta_{b}+\mathrm{dh}_{ref. , b}$ and ${{dr}_{b}=d}_{b}\cos\theta_{b}+\mathrm{dr}_{ref. , b}$.

1. **Experimental methods**

**Preparation of living cells and synthetic vesicles**

The U2OS, HUVECs, and IMR-90 cell lines were purchased from ATCC and maintained in an incubator with 5% CO_2_ at 37°C. Complete culture media suggested by ATCC for each of these cell lines was used.

Lipids and DiI fluorescent dye were purchased from Avanti and Molecular Probes, respectively. The following lipid composition was used for vesicles in phosphate-buffered saline (PBS): 95–92% for POPC, 2 or 5% for 16:0 Biotinyl Cap PE (i.e., biotin lipid), 1% for DiI, and 2% for 16:0 DNP PE. Vesicles were made by following a typical extrusion protocol^39,40^. Here, polycarbonate membrane filters with 100 nm pores purchased from Avanti were used.

**Atomic force microscopy imaging for vesicles**

Atomic force microscopic (AFM) images for the vesicle in the PBS solution were acquired with a NanoWizard Ultra Speed (JPK Instruments) in Quantitative Imaging mode (QI^TM^ parameters: 220–345 pN setpoint, 100–88 nm z-length, 5.5–6 ms pixel time for imaging vesicles in the low avidin condition, and 215 pN setpoint, 66 nm z-length, 5.5 ms pixel time in the high avidin condition). Triangular Si_3_N_4_ cantilevers with a spring constant of 0.02 N/m (OMCL-TR400PSA-1, Olympus) were used.

**Magnetic tweezer experiments for living cells and vesicles**

A magnetic tweezer apparatus built on an inverted microscope (Olympus, IX73) as previously described^9,10^ was used. Here, pre-calibrated forces can be applied by modulating the height of a pair of magnets from samples.

For the magnetic tweezer experiment with living cells, channel slides purchased from ibidi (µ-Slide I Luer) were used. Before culturing cells on the slides, polystyrene reference beads (Spherotech, 3.3um) were non-specifically attached to the bottom surface of the channel slides. Cells were seeded and cultured until they formed an epithelial monolayer on the substrate. Then, streptavidin-coated magnetic beads (Dynabeads® M-280 streptavidin) were introduced to the upper surface of the cellular layer after the treatment and washing of the biotin lipid. Here, 410 μg of biotin lipid was dissolved in 600 μl DMSO, and the cellular layer was incubated for 15–30 min in Live cell imaging solution (LCIS, Molecular Probes®) that contained 0.01–0.04% (v/v) of the lipid-DMSO mixture. The cells were washed with the pure LCIS before the treatment of the magnetic beads. Note that the LCIS was maintained overnight in the incubator with 5% CO_2_ before the magnetic tweezer experiments (as the buffer contains 20 mM HEPSE). 6 μM Latrunculin A (Sigma-Aldrich) was injected together with the magnetic beads for the tubule extraction experiments. For the E-cadherin pulling experiments, biotinylated CD324 (E-Cadherin) monoclonal antibody (DECMA-1) was purchased from eBioscience. Cells seeded in µ-Slide I Luers were treated with ~0.5 ug/ml of DECMA-1 for 30 min and washed before injecting the magnetic beads. For the cholesterol flow experiments, cholesterols and methyl-β-cyclodextrin were purchased from Avanti (#700000P) and Sigma-Aldrich, respectively. A sufficient amount of cholesterol powders was mixed with 20 mM methyl-β-cyclodextrin. After overnight incubation at 37 ℃ with vortexing, undissolved cholesterol remains were filtered from the buffer. The cholesterol-MβCD buffer (150–200 ul) was applied with a flow velocity of 3.3 ul/sec during the buffer exchange processes. Few cycles hardly fitted with the model after the buffer flow were not sampled for some measurements. For all data after the cholesterol-MβCD flow, force vs. extension cycles were continued until the number of cycles taken is greater than two times of the number of cycles that are not sampled. For all live cell experiments, the prepared sample slides were effectively sealed to inhibit any undesired flow of the solution.

For the vesicle experiments, chambers consisting of a channel of ~20 μl volume were constructed by attaching two coverslips with double-sided tape (24 × 40 mm for top and 24 × 50 mm for bottom). Here, the bottom coverslip was coated with polyethylene glycol (PEG) polymer chains. Two types of PEG-coated coverslips were prepared. The first was made by mixing the PEG polymer chains and biotin-conjugated PEG (biotin PEG) polymer chains with a ratio of 200 : 1 (i.e., PEG : biotin PEG = 200 : 1). The other used a PEG : biotin PEG ratio of 10 : 1 (i.e., PEG : biotin PEG = 10 : 1). Note that the ratio of 100:1 and 10:1 were used for AFM imaging of the low-avidin and high-avidin vesicles respectively. The polyethylene glycol (PEG) polymer chains were purchased from Laysan Bio, Inc.

Given the chambers, the vesicle samples were prepared as follows: 1) inject Neutravidin (Invitrogen) 5–0.5 μg/ml, incubate for 5 min, wash with PBS; 2) inject avidin-coated polystyrene ­­beads (Polysciences, 1 μm), incubate for 5 min, wash with PBS; 3) inject the vesicles, incubate for 20 min, wash with PBS; 4) inject anti-dinitrophenol (DNP) antibody-coated magnetic beads, incubate for 15–30 min (for the 200 : 1 slides) or 30–60 min (for the 10 : 1 slides). Here, the anti-DNP antibody (Abcam) was conjugated to the magnetic beads (Dynabeads® M-270 Carboxylic Acid) by following a protocol provided by the manufacturer. Both vesicle and live-cell experiments were performed at room temperature (22–25℃).

1. **Energy barrier analysis**

**Kinetic information from pulling experiments**

To extract information about the energy barrier associated with the data in Fig. 3, a kinetic theory introduced by Dudko et al.^22^ was used. According to this theory, 1) the distance to the transition energy barrier Δx^ǂ^, 2) the height of the barrier ΔG^ǂ^, and 3) the intrinsic rate coefficient for the transition k, can be estimated by fitting the model to the force distribution for the transition p(f) and corresponding force-dependent transition kinetic rate k(f). Here, k(f) can be estimated from the force distribution histogram p(f) through the following equation (26)^41^:

$k_{i}\left( f \right)=\frac{p_{i}r}{\left( \frac{p_{i}}{2}+\sum_{i+1}^{N} p_{i} \right)\Delta}$ (26)

where p_i_, r, and Δ are the probability of the i^th^ bin of the histogram, the constant loading rate in experiments, and the bin width of the histogram, respectively. Note that f, which indicates the change of the generalized force for the middle partition of the vesicle in this case, was approximated with the measured magnetic tip force. Model calculations suggest that changes of the tip force and the middle partition force are approximately equal within the force range examined.

Now, equations (27) and (28) from Dudko et al.^22^ can be fitted onto the transition kinetic rate plot and the transition-force histogram, respectively.

$k\left( f \right)=k_{0}\left( 1-\frac{f\Delta x^{\ddagger}}{\Delta G^{\ddagger}}\nu\right)^{\frac{1}{\nu}-1}\exp\left\{ \beta\Delta G^{\ddagger}\left[ 1-\left( 1-\frac{f\Delta x^{\ddagger}}{\Delta G^{\ddagger}}\nu\right)^{\frac{1}{\nu}} \right] \right\}$ (27)

and

$p\left( f \right)\propto\frac{k\left( f \right)}{r}\exp\left\{ \frac{k_{0}}{\beta\Delta x^{\ddagger}r}-\frac{k\left( f \right)}{\beta\Delta x^{\ddagger}r}\left( 1-\frac{f\Delta x^{\ddagger}}{\Delta G^{\ddagger}}\nu\right)^{1-\frac{1}{\nu}} \right\}$ (28)

where β=1/(k_b_T); k_b_ and T are Boltzmann constant and temperature in Kelvin respectively. Here, Δx^ǂ^, ΔG^ǂ^, and k_0_ are the parameters to be estimated and represent the distance to the energy barrier, the height of the barrier, and the transition kinetic rate when the force change is zero, respectively. The value for v can be chosen from 1/2 to 2/3 depending on the shape of the desired free energy landscape.

**Kinetic information from relaxation experiments**

The theory originally developed for pulling experiments can be equally applied to characterize energy barrier information during relaxation. In doing so, the energy shifted by force f_r_ as the initial reference energy state can be defined first. Then, a situation can be considered in which force is applied (with a constant loading rate) in a direction opposite the reaction coordinate, equivalent to the constant rate of relaxation from force f_r_. With these two assumptions, Dudko’s theory for pulling can be equally applied to the relaxation experiments by simply replacing k_0_ in equations (27) and (28) to k_fr_ and f to f_r_ - f. Here, k_fr_ indicates the intrinsic rate coefficient for the re-transition when the reference force is f_r_. Equations (26)–(28) were used to estimate the energy barrier information for the relaxation.

If i=1, $k_{i}=\frac{p_{i}r}{\left( \frac{p_{i}}{2} \right)\Delta}$ otherwise, $k_{i}=\frac{p_{i}r}{\left( \frac{p_{i}}{2}+\sum_{j=1}^{i-1} p_{j} \right)\Delta}$ (26)

$k\left( f \right)=k_{f_{r}}\left( 1-\frac{\left( f_{r}-f \right)\Delta x^{\ddagger}}{\Delta G^{\ddagger}}\nu\right)^{\frac{1}{\nu}-1}\exp\left\{ \beta\Delta G^{\ddagger}\left[ 1-\left( 1-\frac{\left( f_{r}-f \right)\Delta x^{\ddagger}}{\Delta G^{\ddagger}}\nu\right)^{\frac{1}{\nu}} \right] \right\}$ (27)

and

$p\left( f \right)\propto\frac{k\left( f \right)}{r}\exp\left\{ \frac{k_{f_{r}}}{\beta\Delta x^{\ddagger}r}-\frac{k\left( f \right)}{\beta\Delta x^{\ddagger}r}\left( 1-\frac{\left( f_{r}-f \right)\Delta x^{\ddagger}}{\Delta G^{\ddagger}}\nu\right)^{1-\frac{1}{\nu}} \right\}$ (28)

Finally, for the data in Fig. 3, fitting the force distribution of the transition to the theory gave 19 nm and 30 k_B_T as the distance and height of the energy barrier (at nearly zero tension). The fitting of the retransition force distribution indicated an energy barrier with a distance and height of 15 nm and 33 k_B_T. Here, the reference force value was set at 9 pN of magnetic force for the following reasons. According to the model calculation, the transition state becomes the global minimum at ~9 pN. In addition, the peak of the measured re-transition forces was smaller than that of the transition forces. This suggests that the transited state in stretching can be maintained until the force is significantly reduced in relaxation.

| **Inputs:** a reference curve (from solution of k-1^th^ step), prescribed boundary values  **Main outputs:** solution for nodal DOFs  Re-discretize the finite element nodes from the reference curve  Define the normal directions  Update $d_{1}$= 0  Set j=1  **repeat**  Compute $J\left( d_{j} \right)$ and $G\left( d_{j} \right)$  Update $d_{j+1}$  Set j = j+1  **until** norm($d_{j}$ - $d_{j-1}$) < TOL  compute $f_{0}\left( d_{j} \right)$  save $d_{j}$ and $f_{0}$ |
| --- |

**Algorithm S1. Newton’s methods with a fixed boundary value (for k^th^ step)**

|  | POPC vesicles | Plasma membrane of living cells^(^**^a^**^)^ |
| --- | --- | --- |
| ϕ_0_ (resting lipid area density) | 1000/629 x 10^18^ /m^2^ | 1000/629 x 10^18^ /m^2^ |
| k_m_ (lipid bilayer bending modulus) | 10 k_b_T | 20 k_b_T |
| σ_0_ (lipid bilayer surface tension with zero density strain) | exp(-7) mN/m | exp(-10) mN/m^(^**^b^**^)^ |
| K_app_ (lipid bilayer apparent area stretching modulus) | 150 mN/m | 150 mN/m |

**Table S1. Summary of the membrane parameters**^16,42-44^. (**a**) For the sake of simplicity, these values were used for all cell membrane calculations throughout this study. (**b)** A higher level of surface undulation (excess area) is assumed for cell membranes^45^.

**
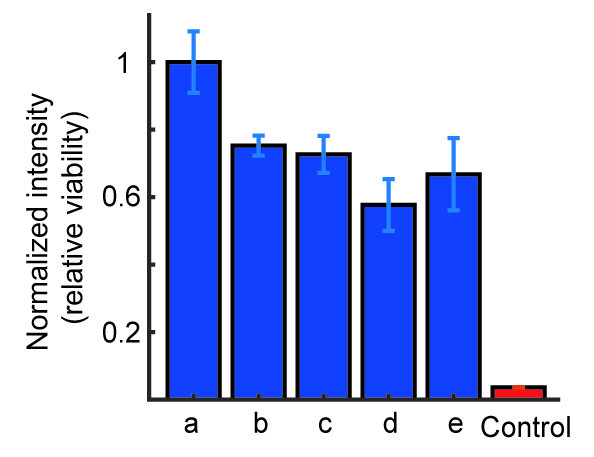
**

**Figure S1. Viability test (MTS assay) for U2OS cells with experimental treatments.** (**a**) One hour in cell culture media, three hours in 5/6 of Live Cell Imaging Solution (LCIS, Molecular Probes®) supplemented with 1/6 of Tetrazolim Salt Solution (TSS, Promega®). (**b**) One hour in LCIS, three hours in LCIS (5/6) + TSS (1/6). (**c**) Thirty minutes in LCIS with 0.01 v/v% lipid-DMSO, 30 minutes in LCIS, three hours in LCIS (5/6) + TSS (1/6). (**d**) Thirty minutes in LCIS with 0.1 v/v% lipid-DMSO, 30 minutes in LCIS, 3 hours in LCIS (5/6) + TSS (1/6). (**e**) Thirty minutes in LCIS with 0.01 v/v% lipid-DMSO, 20 minutes in LCIS, 10 minutes in LCIS with 0.5 μg/mL Latrunculin-A, 3 hours in LCIS (5/6) + TSS (1/6). (**Control**) Dead cells.

**
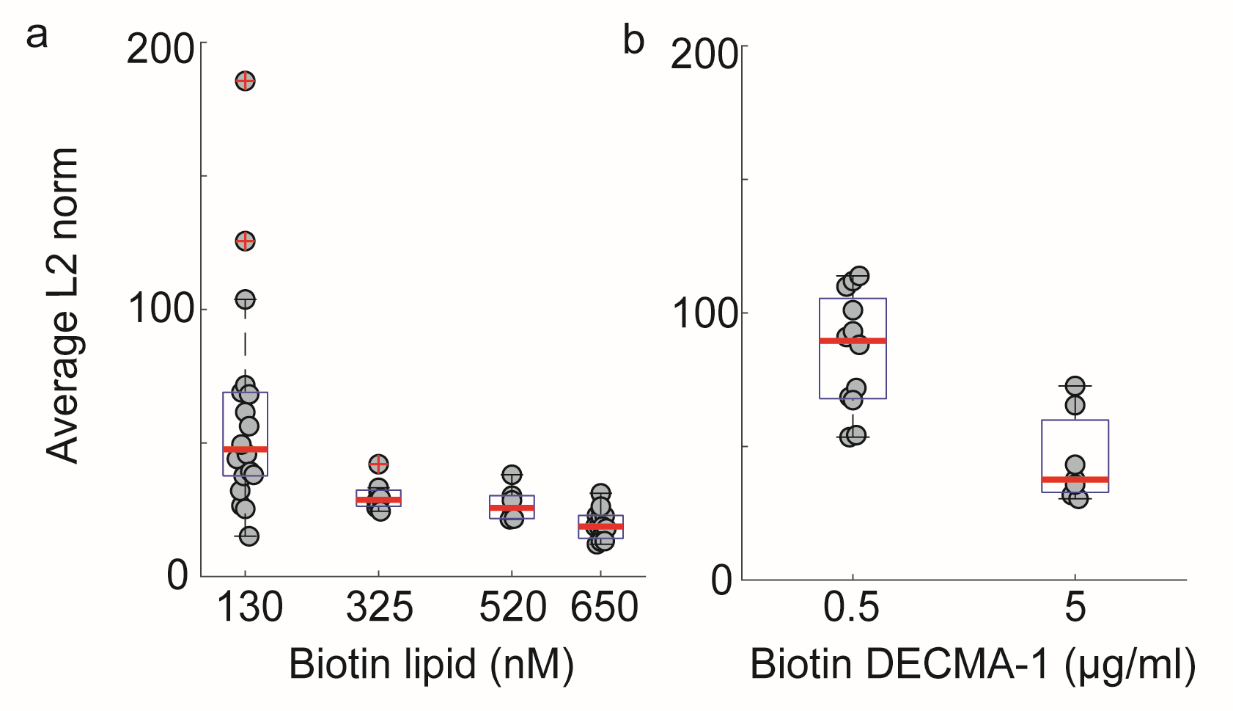
**

**Figure S2. Bead fluctuation characterization.** (**a**) For different amounts of biotin-conjugated lipids applied to the cells, the fluctuation of the magnetic bead is characterized by its L_2_ norm. Euclidean norm of magnetic bead locations with respect to their center point was calculated. Trajectories for a bead were measured for three seconds, and their norm values were averaged. (**b**) The fluctuation of the beads is similarly characterized for the cells treated with biotinylated E-Cadherin monoclonal antibody DECMA-1. Using 0.5 μg/ml of biotin DECMA-1, the fluctuation of the beads on the cell surface was smaller than that using 130 nM biotin-lipids.

­­­
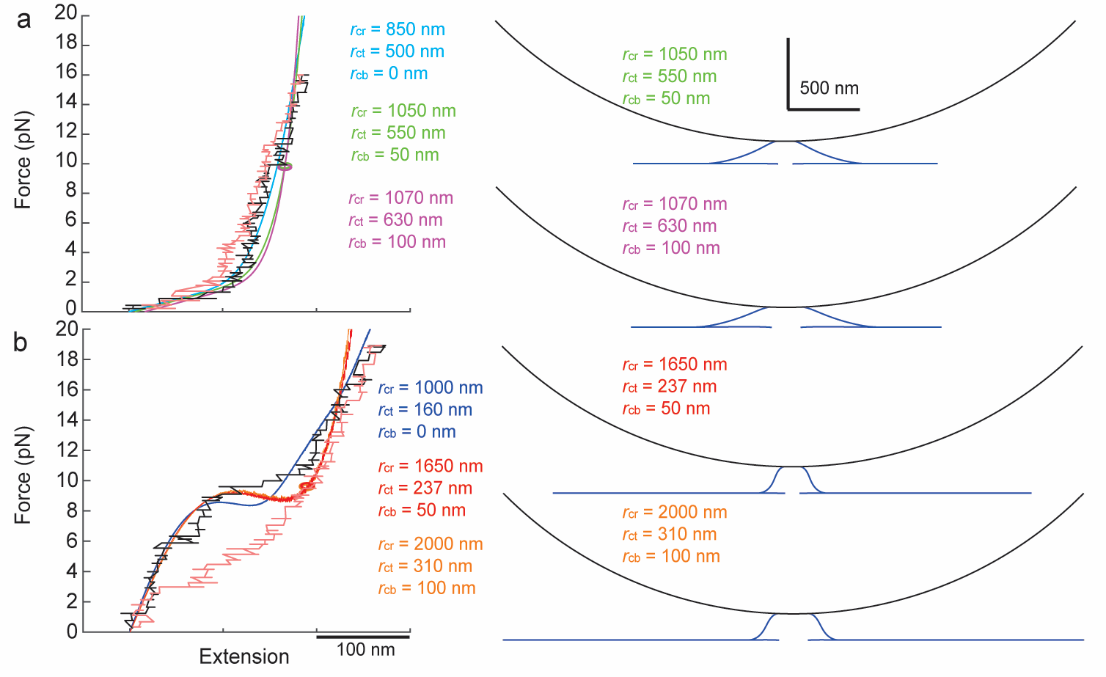


**Figure S3. Calculation of force vs. extension curves with the assumption of non-point-like attachment for magnetic beads.** (**a**) Using the membrane model, a Type I force vs. extension measurement was fitted with different r_ct_ and r_cr_ sets by assuming non-point-like contact between the bead and the membrane. The size of the bead attachment was parameterized with r_cb_. The blue curve assumed the point-like attachment (i.e., r_cb_=0 nm). Membrane shapes that correspond to initial and marked (circles) configurations are shown for r_cb_= 50 nm and r_cb_ =100 nm cases. The diameter of the magnetic bead is 2800 nm. (**b**) The fifth cycle of Fig. 1e was compared with the model using different r_ct_ and r_cr_ sets. The dark blue curve copied from Fig. 1e assumed the point-like attachment. For both Type I in **a** and Type II in **b**, the greater the value assumed for r_cb_, the greater the values for r_ct_ and r_cr_ that were required to fit the measurement. For the sake of simplicity (i.e., reduced number of parameters used in the model), the additional free parameter r_cb_ was not defined in the other places of the manuscript.

**
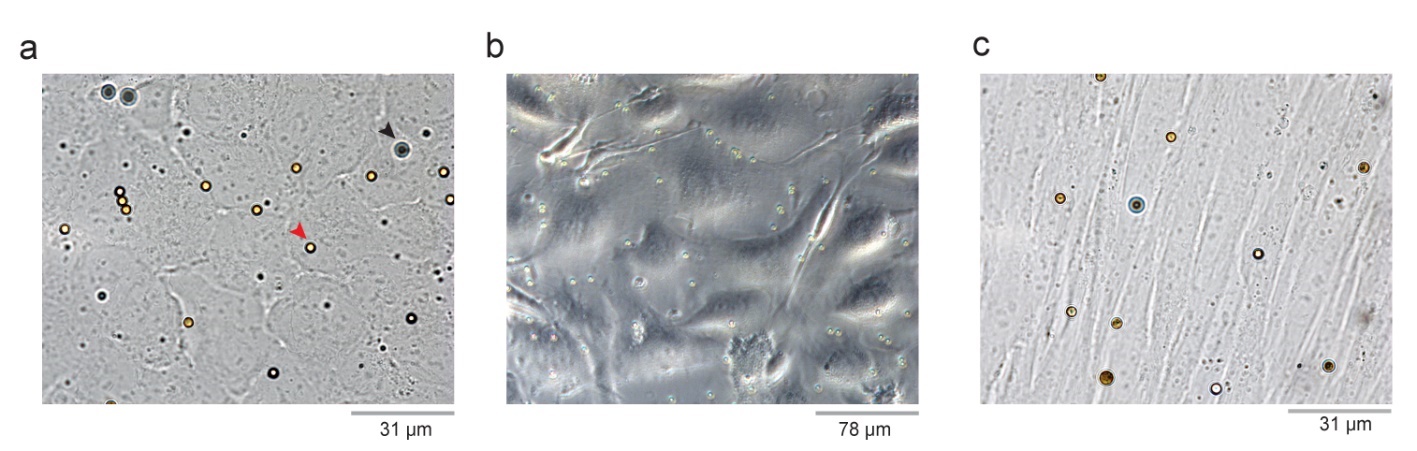
**

**Figure S4. Bright-field images for live-cell samples.** (**a**) U2OS cells imaged in differential interference contrast (DIC) mode (red arrow: magnetic bead, black arrow: reference bead). (**b**) HUVEC cells imaged in phase-contrast mode. (**c**) IMR 90 cells imaged in DIC mode.

**
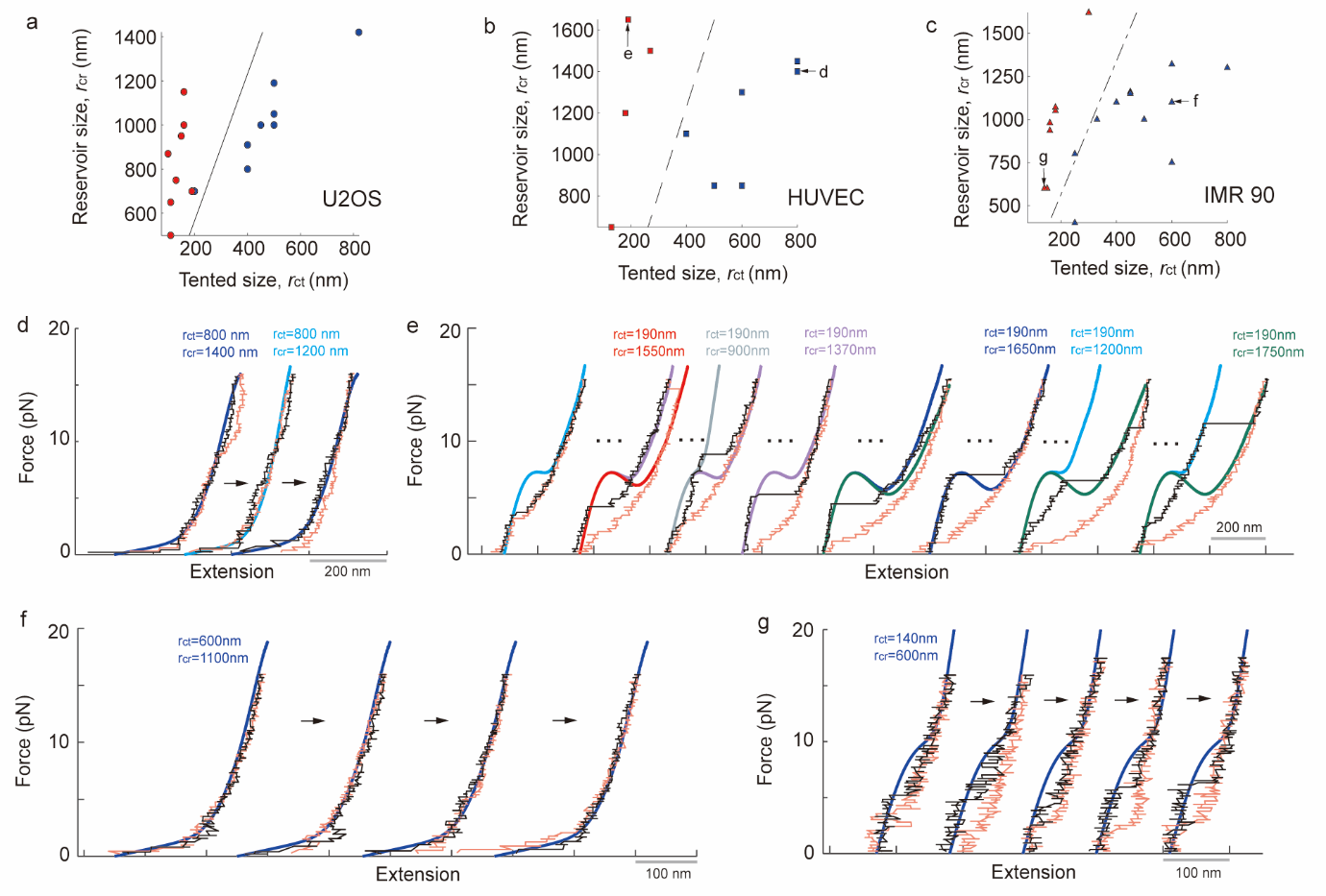
**

**Figure S5. Individual r_ct_ - r_cr_ diagram for three different cell lines.** (**a**) Diagram for U2OS cells. (**b**) Diagram for HUVECs. (**c**) Diagram for IMR-90 cells. A merged diagram for these three cell lines is shown in Fig. 1i. (**d**) Type I force vs. extension traces obtained from HUVECs (successive measurements from a single bead). (**e**) Measurements from the HUVECs (non-successive from a single bead) showing Type II responses. Note that a small transition that can be fitted with a calculation (gray) was identified for the third force vs. extension measurement. The initial force barrier in the seventh response was continuous (though it was not directly fitted) with the larger extension regime of a calculation using r_ct_ = 190 nm, r_cr_ = 1200 nm (blue). (**f**) Type I force vs. extension traces obtained from IMR 90 cells. (**g**) Type II traces obtained from IMR 90 cells

**
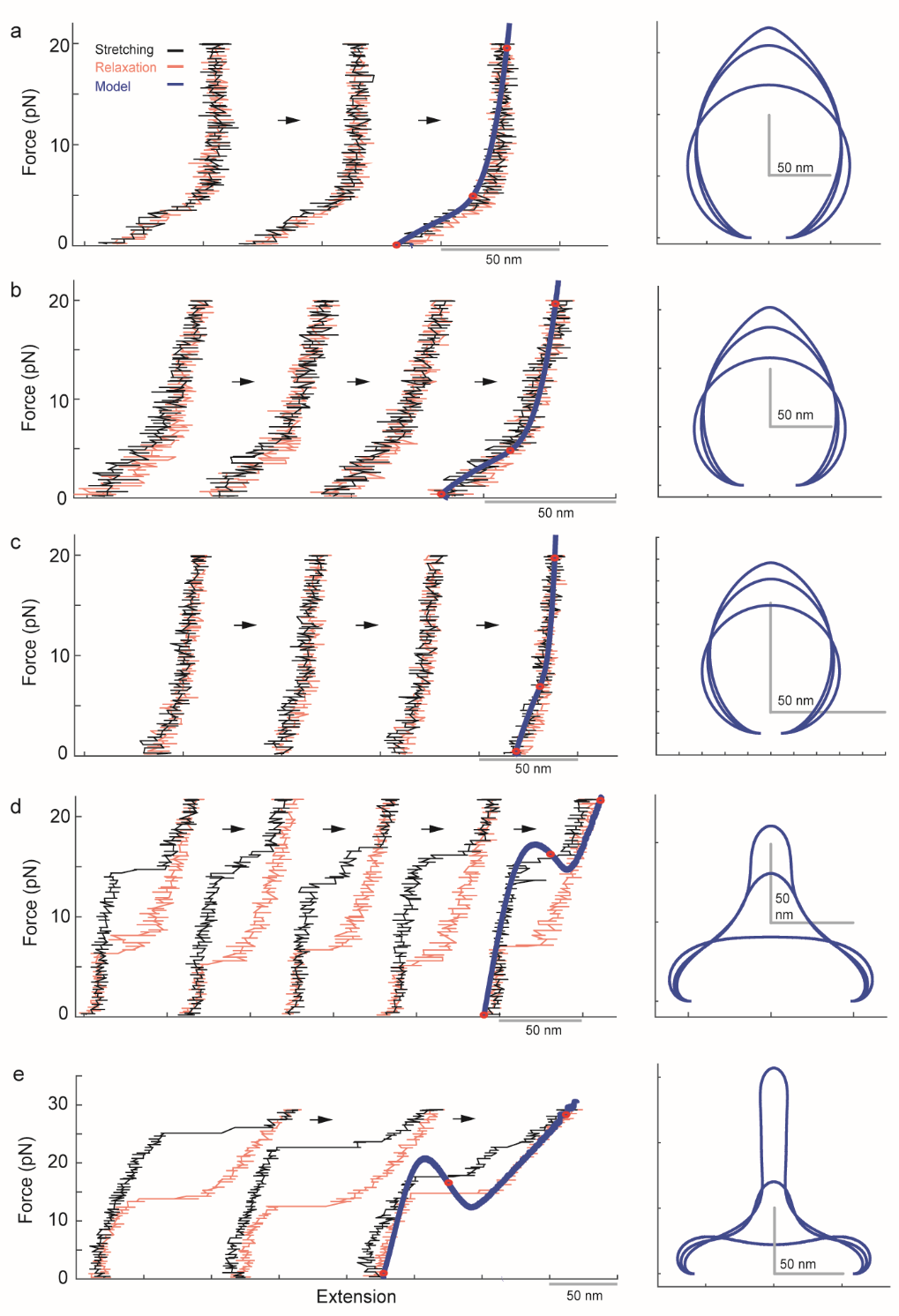
**

**Figure S6. Nanovesicle force vs. extension curves.** (**a**-**c**) Observed in the low avidin condition. (**d**, **e**) Observed in the high avidin condition. The calculations used r_vr_ = 65 nm, r_vc_ = 15 nm for **a**; r_vr_ = 60 nm, r_vc_ = 22 nm for **b**; r_vr_ = 30 nm, r_vc_ = 5 nm for **c**; r_vr_ = 50 nm, r_vc_ = 49 nm for **d**; and r_vr_ = 55 nm, r_vc_ = 59 nm for **e**.

**
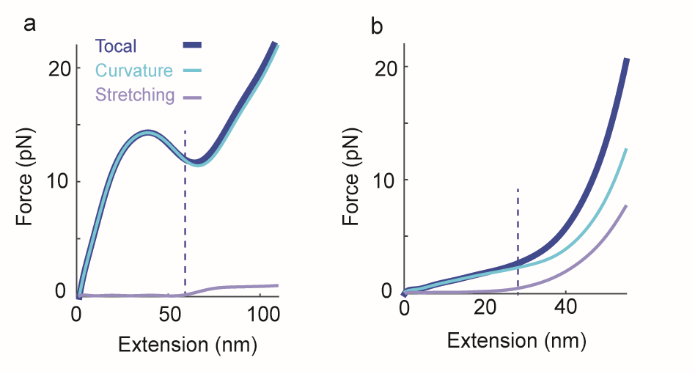
**

**Figure S7. Force decomposition analysis.** (**a**) The calculation in Fig. 2e (dark blue) is decomposed into two different force contributions based on the respective energy terms in equation (2). The force vs. extension curve in light blue is responsible for the membrane curvature energy (curvature force), while the curve in purple is responsible for the area strain energy (area-strain force). (**b**) Similar analyses were performed for the calculation in Fig. 2f. For both **a** and **b**, the mean and area strain energies were post calculated from the finite element solutions, and the energy vs. extension curves were fitted with the fourier8 function in Matlab to get the curvature and area-strain forces.

­


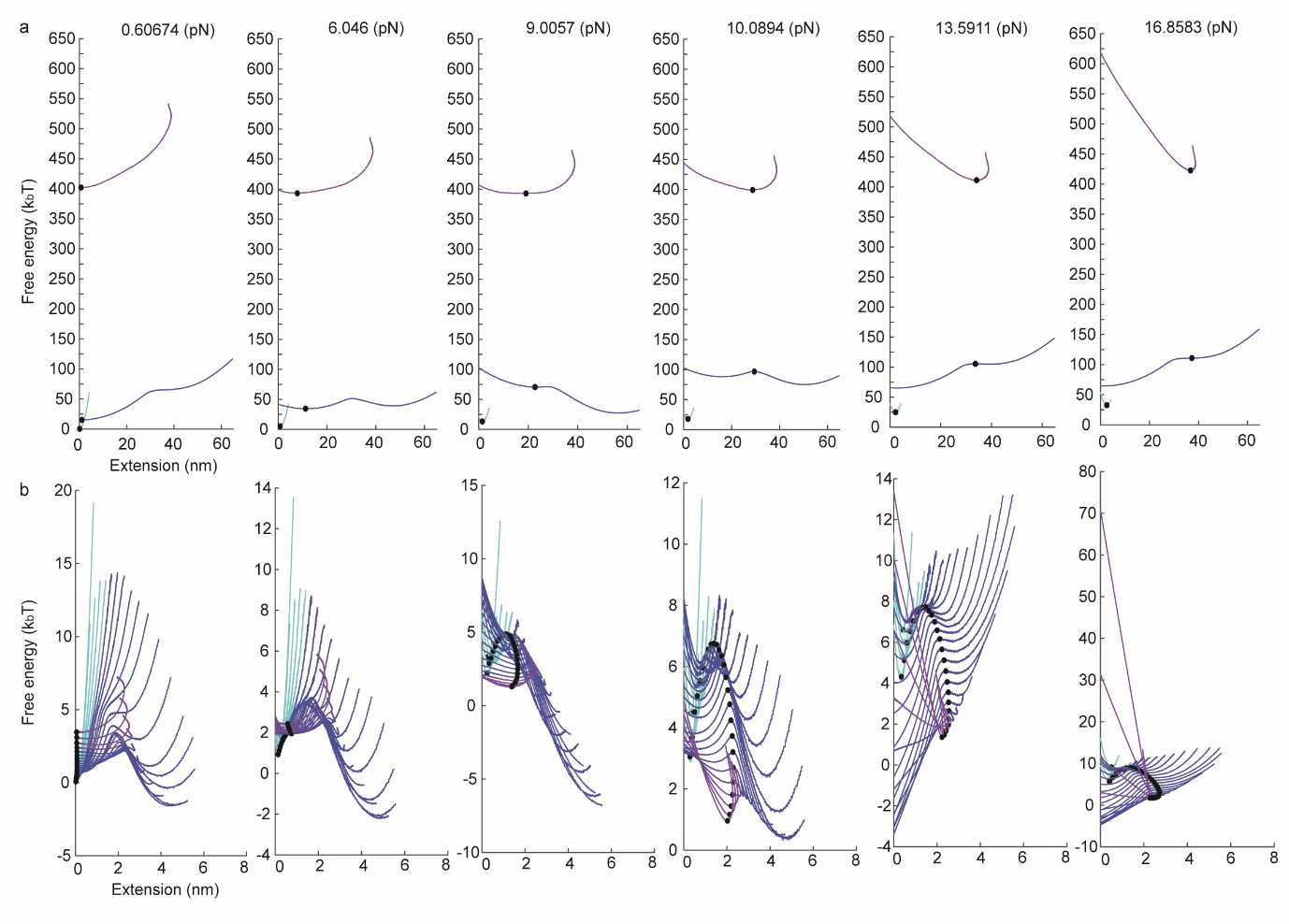


**Figure S8. Detailed free energy calculation for the vesicle in Fig. 3.** (**a**) Energy calculations for the three membrane partitions in Fig. 3b (blue, dark blue, purple for tip, middle, bottom partitions, respectively) with respect to different levels of vesicle tip force. Black dots indicate a stationary point that corresponds to calculated shape of the vesicle in Fig. 3b. (**b**) Finer sectioning of each of the three partitions and their corresponding free energies. Blue, dark blue, and purple are sections from tip, middle, bottom partitions, respectively. Only four sections in the bottom partition were plotted. Black dots indicate the stationary state of each section.

**
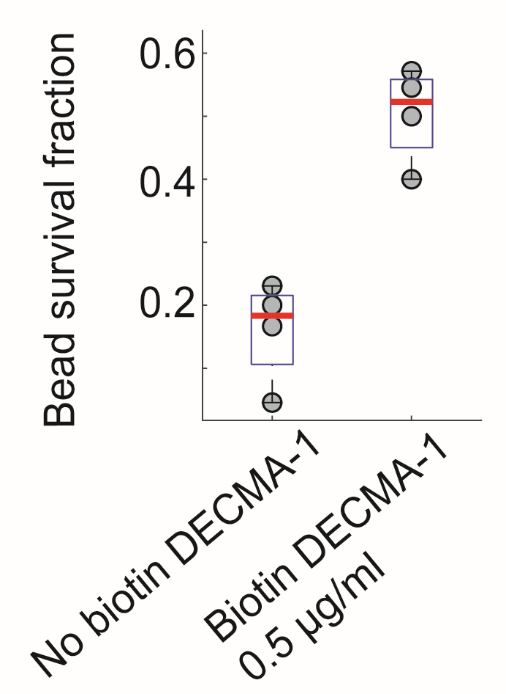
**

**Figure S9. Magnetic bead targeting to the cell surface via biotinylated E-Cadherin antibody.** Magnetic bead survival fraction (with ~13 pN in ~15 seconds) with the treatment of 0.5 μg/ml of biotinylated E-Cadherin monoclonal antibody DECMA-1 to the cells. About half of the injected beads were still bound to the surface after the force application with the treatment of 0.5 μg/ml biotinylated DECMA-1.

**
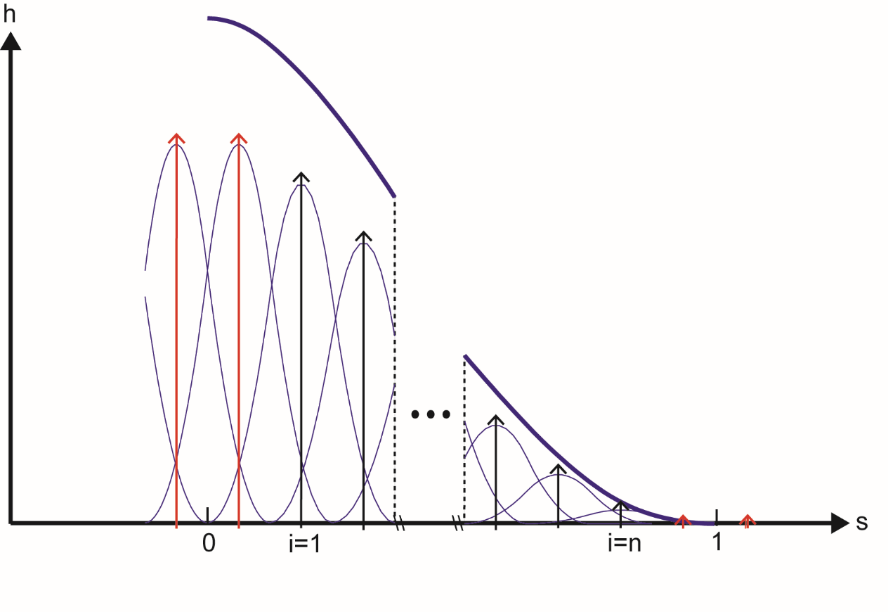
**

**Figure S10. Description of B-spline basis functions and nodal values for the finite element model.** Membrane shape function h(s) and B-spline basis functions are shown with bold blue and thin blue curves, respectively. Black arrows indicate nodal values (from i = 1 to i = n) associated with their B-spline functions. Red arrows indicate fixed values that define boundary conditions. Shape function r(s) is defined identically.

**Additional references**

41 Dudko, O. K., Hummer, G. & Szabo, A. Theory, analysis, and interpretation of single-molecule force spectroscopy experiments. *Proceedings of the National Academy of Sciences* **105**, 15755-15760 (2008).

42 Petrache, H. I., Dodd, S. W. & Brown, M. F. Area per lipid and acyl length distributions in fluid phosphatidylcholines determined by 2H NMR spectroscopy. *Biophysical journal* **79**, 3172-3192 (2000).

43 Dimova, R. Recent developments in the field of bending rigidity measurements on membranes. *Advances in colloid and interface science* **208**, 225-234 (2014).

44 Evans, E., Heinrich, V., Leung, A. & Kinoshita, K. Nano-to microscale dynamics of P-selectin detachment from leukocyte interfaces. I. Membrane separation from the cytoskeleton. *Biophysical journal* **88**, 2288-2298 (2005).

45 Morris, C. & Homann, U. Cell surface area regulation and membrane tension. *The Journal of membrane biology* **179**, 79-102 (2001).
